# A Novel Flexible Inertia Weight Particle Swarm Optimization Algorithm

**DOI:** 10.1371/journal.pone.0161558

**Published:** 2016-08-25

**Authors:** Mohammad Javad Amoshahy, Mousa Shamsi, Mohammad Hossein Sedaaghi

**Affiliations:** Department of Electrical Engineering, Sahand University of Technology, Tabriz, Iran; Southwest University, CHINA

## Abstract

Particle swarm optimization (PSO) is an evolutionary computing method based on intelligent collective behavior of some animals. It is easy to implement and there are few parameters to adjust. The performance of PSO algorithm depends greatly on the appropriate parameter selection strategies for fine tuning its parameters. Inertia weight (IW) is one of PSO’s parameters used to bring about a balance between the exploration and exploitation characteristics of PSO. This paper proposes a new nonlinear strategy for selecting inertia weight which is named Flexible Exponential Inertia Weight (FEIW) strategy because according to each problem we can construct an increasing or decreasing inertia weight strategy with suitable parameters selection. The efficacy and efficiency of PSO algorithm with FEIW strategy (FEPSO) is validated on a suite of benchmark problems with different dimensions. Also FEIW is compared with best time-varying, adaptive, constant and random inertia weights. Experimental results and statistical analysis prove that FEIW improves the search performance in terms of solution quality as well as convergence rate.

## 1 Introduction

Swarm intelligence is an exciting new research field still in its infancy compared to other paradigms in artificial intelligence [1]. One of the research areas within computational swarm intelligence is particle swarm optimization (PSO), which developed by Eberhart and Kennedy in 1995 [2, 3], inspired by intelligent collective behavior of some animals such as flocks of birds or schools of fish. In PSO, each individual represents a potential solution and is termed as “particle” and the flock of particles called “swarm” represents the population of individuals, so a population of potential solutions is evolved through successive iterations. The most important advantages of the PSO, compared to other optimization strategies, lies in its speedy convergence towards global optimum, easily implementable code, complex computation free environment and having few parameters to adjust. Accelerating convergence speed and avoiding the local optima have become the two most important and appealing goals in PSO research. A number of variant PSO algorithms have, hence, been proposed to achieve these two goals [4, 5]. It is seen to be difficult to simultaneously achieve both goals. For example, the comprehensive-learning PSO in [5] focuses on avoiding the local optima, but brings in a slower convergence as a result. Therefore, despite being having several attractive features and a potential global optimizer, PSO alike several other populations based search algorithms have certain drawbacks associated with it. To overcome the drawbacks caused by “stagnation of particles”, several attempts have been made to enhance the performance of PSO and the improved variants superseded the standard one. Some of these include, proposing inertia weight (IW) [6, 7], introducing constriction factor based PSO [8], weighting particle’s own experience and neighbors experience [9], fine tuning of various PSO parameters [10], proposing different interaction methods among PSO particles [11, 12]. Moreover PSO has been hybridized [13] with concepts borrowed from other heuristic and deterministic algorithms to improve its searching ability and enhancing its convergence towards global optima. As we know, IW can balance the proportion of global search ability and local exploration ability. When its value is bigger, the algorithm has a stronger global search ability and poorer local exploration ability. When IW value is smaller, global search ability and local exploration ability are just reverse. In the other word, IW controls the particle’s momentum and so many strategies have been proposed in previous studies to choose a suitable IW that maintains the exploration–exploitation trade-off throughout the searching process. In this paper we propose a flexible exponential inertia weight (FEIW) PSO algorithm (FEPSO) for optimization problems. This work differs from the existing time-varying IW strategies at least in two aspects: firstly, it proposes a flexible IW, which can adapt with each problem, i.e., for a certain optimization problem, with suitable parameter selection, we can get a special IW strategy that has best performance for solving it. The second is to compare the best time-varying, adaptive and primitive IW strategies with FEIW and obtain that FEPSO is more efficacious for optimization problem.

The rest of this paper is organized as follows: Section ‎2 presents the principles of particle swarm optimization algorithm. A review on inertia weight strategies is stated in section ‎3. Proposed inertia weight and its properties will be discussed in section ‎4. In Section ‎5, parameter settings and performance evaluation criteria is introduced. The numerical analysis, statistical tests and discussion of results is performed under section ‎6 and the conclusions are given in section ‎7.

## 2 The Principles of Particle Swarm Optimization Algorithm

The basic idea of the PSO algorithm is to search out the optimum value by collaborating and sharing information between the individuals, and the particle’s quality could be measured according to the fitness value of particles. First, the positions and velocities of a group of particles are initialized randomly, and then the optimal solution can be searched out by updating generations in the search space. Suppose that the size of the swarm is *M* and the search space is *D* − dimensional. The position of the *i*th particle is presented as *x*_*i*_ = (*x*_*i*1_, *x*_*i*2_, …, *x*_*iD*_) where *x*_*id*_ ∈ [*l*_*d*_, *u*_*d*_], *d* ∈ [1, *D*], and *l*_*d*_ and *u*_*d*_ are the lower and upper bounds of the *d*th dimension of the search space. The velocity of each particle is represented with a vector. The *i*th particle velocity is presented as *v*_*i*_ = (*v*_*i*1_, *v*_*i*2_, …, *v*_*iD*_). At each time step, the position and velocity of the particles are updated according to the following equations [2]:

$$\nu_{ij}(t+1)=\nu_{ij}(t)+\;c_{1}\;r_{1ij}\;[p_{bestij}(t)-x_{ij}(t)]\;+\;c_{2}\;r_{2ij}\;[g_{bestj}(t)-x_{ij}(t)]\;$$ (1)

$$x_{ij}(t+1)=x_{ij}(t)+\nu_{ij}(t+1)$$ (2)

where *r*_1*ij*_, *r*_2*ij*_ are two distinct random numbers [2], generated uniformly from the range [0,1], the acceleration coefficients *c*_1_, *c*_2_ are two positive constants [3] and *t* is the current iterative time. The best previous position found so far by this particle is denoted as *p*_*besti*_ = (*p*_*i*1_, *p*_*i*2_, … ,*p*_*iD*_), and the best previous position discovered by the whole swarm is denoted as *g*_*best*_ = (*g*_1_, *g*_2_, … ,*g*_*D*_). The velocity of particle should be under the constrained conditions [*v*_min_, *v*_max_]^*D*^.

The balance between global and local search throughout the course of a run is critical to the success of an optimization algorithm [14]. Almost all of the evolutionary algorithms utilize some mechanisms to achieve this goal. To bring about a balance between the exploration and exploitation characteristics of PSO, Shi and Eberhart proposed a PSO based on inertia weight (*ω*) in which the velocity of each particle is updated according to the following equation [15]:

$$\nu_{ij}(t+1)=\omega \nu_{ij}(t)+\;c_{1}\;r_{1ij}\;[p_{bestij}(t)-x_{ij}(t)]\;+\;c_{2}\;r_{2ij}\;[g_{bestj}(t)-x_{ij}(t)]\;$$ (3)

They claimed that a large IW facilitates a global search while a small IW facilitates a local search. By changing the IW dynamically, the search capability is dynamically adjusted. This is a general statement about the impact of *ω* on PSO’s search behavior shared by many other researchers. However, there are situations where this rule cannot be applied successfully [16].

The PSO procedure can be divided into the following steps:

1. Initialize the original position and velocity of particle swarm;
2. Calculate the fitness value of each particle;
3. For each particle, compare the fitness value with the fitness value of *p*_*best*_, if current value is better, then renew the position with current position, and update the fitness value simultaneously;
4. Determine the best particle of group with the best fitness value, if the fitness value is better than the fitness value of *g*_*best*_, then update the *g*_*best*_ and its fitness value with the position;
5. Check the finalizing criterion, if it has been satisfied, quit the iteration;
6. Update the position and velocity of particle swarm, return to step 2.

## 3 Review on Inertia Weight Strategies

Since the initial development of PSO, several variants of this algorithm have been proposed by researchers. The basic PSO, presented by Kennedy and Eberhart in 1995 [2], has no IW. The first modification introduced in PSO was the use of an IW parameter in the velocity update equation of the initial PSO resulting in Eq (3), a PSO model which is now accepted as the global best PSO algorithm [15]. In this section, the various IW strategies are categorized into three classes. The “primitive class” contains strategies in which the value of the IW is constant during the search or is determined randomly. None of these methods uses any input. The “adaptive class” contains those methods which use a feedback parameter to monitor the state of the algorithm and adjust the value of the IW. The “time-varying class” is defined as a function of time or iteration number.

### 3.1 Primitive class

IW parameter was originally introduced by Shi and Eberhart in [15]. They used a range of constant IW (CIW) values

ω=c (4)

and showed that by using large values of *ω*, i.e. *ω* > 1.2, PSO only performs a weak exploration and with low values of this parameter, i.e. *ω* > 0.8, PSO tends to traps in local optima. They suggest that with a *ω* within the range [0.8,1.2], PSO finds the global optimum in a reasonable number of iterations. Shi and Eberhart analyzed the impact of the IW and maximum velocity on the performance of the PSO in [6]. In [17], a random IW (RIW) is used to enable the PSO to track the optima in a dynamic environment.

$$\omega =\frac{1+Rand()}{2}$$ (5)

where *Rand*() is a random number in [0.1]; *ω* is then a uniform random variable in the range [0.5,1].

### 3.2 Adaptive class

Adaptive IW strategies are those that monitor the search situation and adapt the IW value based on one or more feedback parameters. In [18], Arumugam and Rao use the ratio of the global best fitness and the average of local best fitness of particles to determine the IW in each iteration with

$$\omega (t)=1.1-\frac{f\left(g_{best}(t)\right)}{\text{Average}\left(f\left(p_{besti}(t)\right)\right)}$$ (6)

where *f*(.) is the fitness function. The inertia weight in (6) is termed global-average local best IW (GLBIW). Clerc [19] proposes an adaptive inertia weight (AIW) approach where the amount of change in the inertia value is proportional to the relative improvement of the swarm. Let *x*_*i*_(t) denote the position of particle *i* in the search space at time step *t*. The inertia weight is adjusted according to

$$\omega_{i}(t+1)=\omega (0)+\left(\omega (I_{\text{max}})-\omega (0)\right)\times \frac{e^{m_{i}(t)}-1}{e^{m_{i}(t)}+1}$$ (7)

where the relative improvement, *m*_*i*_, is estimated as

$$m_{i}(t)=\frac{f\left(g_{best}(t)\right)-f\left(x_{i}(t)\right)}{f\left(g_{best}(t)\right)+f\left(x_{i}(t)\right)}$$ (8)

with *ω*(*I*_max_) ≈ 0.5 and *ω*(0) < 1.

### 3.3 Time-varying class

Most of the PSO variants use time-varying IW strategies in which the value of the IW is determined based on the iteration number. Time-varying IW strategies have important applications in various fields yet [20, 21]. These methods can be either linear or non-linear and increasing or decreasing. In [8], a linear decreasing IW (LDIW) was introduced and was shown to be effective in improving the fine-tuning characteristic of the PSO. In this method, the value of *ω* is linearly decreased from an initial value (*ω*_max_) to a final value (*ω*_min_) according to the following equation:

$$\omega (t)=\omega_{\text{max}}-t\times \frac{\omega_{\text{max}}-\omega_{\text{min}}}{I_{\text{max}}}$$ (9)

where *t* and *I*_max_ are the current iterative time and the maximum iterative time, respectively. This strategy is very common and most of the PSO algorithms adjust the value of IW using this updating scheme.

Accepting the general idea of decreasing the IW over iterations, some researchers proposed nonlinear decreasing strategies. Chatterjee and Siarry [22] propose a nonlinear decreasing variant of IW in which at each iteration of the algorithm, *ω* is determined based on the following equation:

$$\omega (t)={\left(\frac{I_{\text{max}}-t}{I_{\text{max}}}\right)}^{n}\left(\omega_{\text{max}}-\omega_{\text{min}}\right)+\omega_{\text{min}}$$ (10)

where *n* is the nonlinear modulation index. Different values of *n* result in different variations of IW all of which start from *ω*_max_ and end at *ω*_min_. Feng et al. [23, 24] use a chaotic IW (CHIW) in which a chaotic term is added to the LDIW. The proposed *ω* is as follows.

$$\omega (t)=(\omega_{1}-\omega_{2})\times \frac{I_{\text{max}}-t}{I_{\text{max}}}+\omega_{2}\times z$$ (11)

where *ω*_1_ and *ω*_2_ are the original value and the final value of IW and *z* = 4*z* (1 − *z*). The initial value of *z* is selected randomly within the range(0,1). Chen et al. [25] propose a natural exponential inertia weight (NEIW) strategy according to the following equation:

$$\omega (t)=\omega_{\text{min}}+\left(\omega_{\text{max}}-\omega_{\text{min}}\right)\times e^{-{\left[t/\left(\frac{I_{\text{max}}}{4}\right)\right]}^{2}}$$ (12)

where *ω*_min_ = 0.4 and *ω*_max_ = 0.9, which is found to be very effective for NEIWPSO.

Li and Gao [26] give a kind of exponent decreasing inertia weight (EDIW)

$$\omega (t)=\left(\omega_{\text{max}}-\omega_{\text{min}}-d_{1}\right)e^{\frac{I_{\text{max}}}{I_{\text{max}}+d_{2}t}}.$$ (13)

The massive experiments indicate the algorithm performance can enhance greatly when *ω*_min_ = 0.4, *ω*_max_ = 0.95, *d*_1_ = 0.2 and *d*_2_ = 7. In [27], Bansal et al. implemented a comparative study on fifteen IW strategies to select best IW strategies. With *c* = 7 for CIW, *ω*_min_ = 0.4, *ω*_max_ = 0.9 for LDIW and *ω*_1_ = 0.9, *ω*_2_ = 0.4 for CHIW, They concluded that CHIW is the best strategy for better accuracy and RIW strategy is best for better efficiency. Also it is shown that CIW and LDIW are best inertia weights based on minimum error. Arasomwan and Adewumi [28] established the fact that LDIW is very much efficient if its parameters are properly set. They showed that with good experimental setting, LDIW will perform competitively with similar variants. Thus in this paper, for comparative studies, we use of CIW, RIW, LDIW, CHIW, NEIW, EDIW, GLBIW and AIW as eight well-known primitive, time-varying and adaptive IW strategies.

## 4 Proposed Inertia Weight and Its Properties

In order to overcome the premature convergence, low efficiency or low accuracy of the other IW strategies, we introduce a novel IW strategy for improving the performance of PSO. In this section, first this new IW will be introduced then its properties will be analyzed. At the end, we introduce the IW strategy parameters.

### 4.1 Proposed inertia weight strategy

**Definition.** Suppose *ω*_1_, *ω*_2_ and *ψ* are positive real numbers. We define an inertia weight strategy by

$$\omega (t)=\alpha_{1}e^{\frac{-\psi t}{I_{\text{max}}}}+\alpha_{2}e^{\frac{\psi t}{I_{\text{max}}}}$$ (14)

where

$$\alpha_{1}=\frac{\omega_{2}e^{\psi }-\omega_{1}e^{2\psi }}{1-e^{2\psi }}$$ (15)

$$\alpha_{2}=\frac{\omega_{1}-\omega_{2}e^{\psi }}{1-e^{2\psi }}$$ (16)

and t ∈ [0,*I*_max_] is an integer number. In this strategy, *t* and *I*_max_ are the current iterative time and the maximum iterative time, respectively. The parameters *ω*_1_ and *ω*_2_ are inertia weight at the start and inertia weight at the end of a given run, respectively. In the other word

$$\omega (0)=\omega_{1}\begin{array}{ccc} & \text{and} & \omega_{1}>0 \end{array},$$ (17)

and

$$\omega (I_{\text{max}})=\omega_{2}\begin{array}{ccc} & \text{and} & \omega_{2}>0 \end{array}.$$ (18)

**Fig 1:**
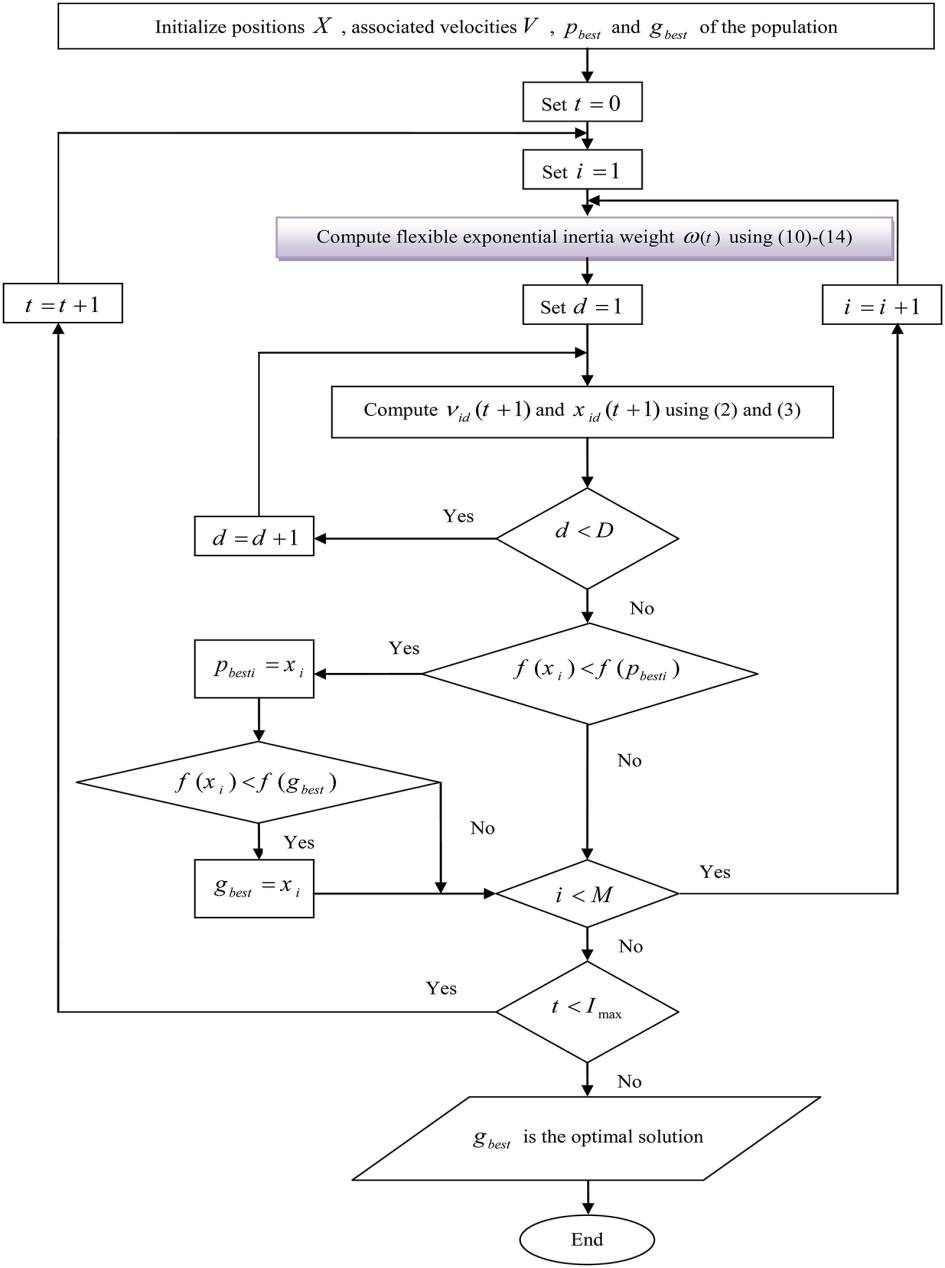
Flow-chart for the proposed technique.

We call *ω*(*t*), the Flexible Exponential Inertia Weight (FEIW) strategy because it can adapt with each problem, i.e., with suitable parameters selection, we can construct many increasing or decreasing inertia weights, or even a lot of strategies with one global minimum in [0,*I*_max_], thus FEIW encompasses a wide range of IW strategies. There is a trade-off between accuracy and efficiency of the PSO algorithm and one of the most important of applications of FEIW is that according to each problem, one can easily change the parameters *ω*_1_, *ω*_2_ and *ψ*, to achieve better accuracy or better efficiency or both of them. Fig 1 shows the flow-chart for PSO based on the FEIW technique used in this paper.

### 4.2 Flexible exponential inertia weight analysis

Before using FEIW, we should have some information about its behavior. In particular, to select its parameters, we need a careful analysis of the function *ω*(*t*) In this subsection, for a mathematical analysis of FEIW, suppose that t ∈ [0,*I*_max_] be a real number instead of integer number. We define a new function by

$$T_{p}(x,y)=y-xe^{p},$$ (19)

and call it as “check function”. Also the notation sgn(.) means the sign function is as follows:

$$\text{sgn}(x)=\{\begin{array}{cc} \frac{x}{\left|x\right|} & x\neq 0\\ 0 & x=0 \end{array}.$$

**Lemma 1.** The check function has the following properties:

$$\text{sgn}(\alpha_{1})=-\text{sgn}\left(T_{\psi }(\omega_{1},\omega_{2})\right)$$ (20)

and

$$\text{sgn}(\alpha_{2})=-\text{sgn}\left(T_{\psi }(\omega_{2},\omega_{1})\right).$$ (21)

**Proof.** According to definition of FEIW, *ψ* > 0 thus 1 –*e*^2*ψ*^ < 0, therefore based on Eq (15),

$$\text{sgn}(\alpha_{1})=-\text{sgn}(\omega_{2}e^{\psi }-\omega_{1}e^{2\psi })=-\text{sgn}(e^{\psi })\text{sgn}(\omega_{2}-\omega_{1}e^{\psi })=-\text{sgn}\left(T_{\psi }(\omega_{1},\omega_{2})\right).$$

Similarly one can prove the other term.

**Lemma 2.** The equation *ω*(*t*) = 0 has at most one root. This equation has a root if and only if

$$\text{sgn}\left(T_{\psi }(\omega_{1},\omega_{2})*T_{\psi }(\omega_{2},\omega_{1})\right)=-1.$$ (22)

In addition, this only root, if it exists, is at $t^{*}=\frac{I_{\text{max}}}{2\psi }\text{ln}\left(\frac{-\alpha_{1}}{\alpha_{2}}\right)$. Also t* ∈ [0,*I*_max_] if and only if

$$\left|\text{ln}\left(\frac{-T_{\psi }(\omega_{1},\omega_{2})}{T_{\psi }(\omega_{2},\omega_{1})}\right)\right|\leq \psi .$$ (23)

**Proof.** By using relation (14), we have

$$\omega (t)=0\Leftrightarrow \alpha_{1}e^{\frac{-\psi t}{I_{\text{max}}}}=-\alpha_{2}e^{\frac{\psi t}{I_{\text{max}}}}\Leftrightarrow \text{ln}\left(\frac{-\alpha_{1}}{\alpha_{2}}\right)=\frac{2\psi t}{I_{\text{max}}}\Leftrightarrow t^{*}=\frac{I_{\text{max}}}{2\psi }\text{ln}\left(\frac{-\alpha_{1}}{\alpha_{2}}\right).$$

From Lemma1 and relation (22), we can conclude $\frac{-\alpha_{1}}{\alpha_{2}}>0$, hence the proof is complete. On the other hand, *ψ* > 0 and *I*_max_ > 0, thus

$$0\leq t^{*}\leq I_{\text{max}}\Leftrightarrow 0\leq \frac{I_{\text{max}}}{2\psi }\text{ln}\left(\frac{-\alpha_{1}}{\alpha_{2}}\right)\leq I_{\text{max}}\Leftrightarrow 0\leq \text{ln}\left(\frac{-\alpha_{1}}{\alpha_{2}}\right)\leq 2\psi \Leftrightarrow 1\leq \frac{-\alpha_{1}}{\alpha_{2}}\leq e^{2\psi }.$$

Using Eqs (15), (16) and (19), we have

$$1\leq -\frac{\omega_{2}e^{\psi }-\omega_{1}e^{2\psi }}{\omega_{1}-\omega_{2}e^{\psi }}\leq e^{2\psi }\Leftrightarrow e^{-\psi }\leq -\frac{\omega_{2}-\omega_{1}e^{\psi }}{\omega_{1}-\omega_{2}e^{\psi }}\leq e^{\psi }\Leftrightarrow -\psi \leq \text{ln}\left(-\frac{T_{\psi }(\omega_{1},\omega_{2})}{T_{\psi }(\omega_{2},\omega_{1})}\right)\leq \psi .$$

**Corollary 1.** For all t ∈ [0,*I*_max_], *ω*(*t*) ≥ 0.

**Proof.** Suppose ∃*t*_0_ ∈ [0,*I*_max_]: *ω*(*t*) < 0. First note that based on relations (17) and (18), the end points of curve of *ω*(*t*) have positive values. Since *ω*(*t*) is a continuous function, thus it has at least two roots, a contradiction, because according to Lemma 2, the equation *ω*(*t*) = 0 has at most one root.

**Corollary 2.** If sgn(*T*_*ψ*_(*ω*_1,_*ω*_2_) * *T*_*ψ*_(*ω*_2,_*ω*_1_)) = 1 then *T*_*ψ*_(*ω*_1,_*ω*_2_) < 0 and *T*_*ψ*_(*ω*_2,_*ω*_1_) < 0.

**Proof.** Let *T*_*ψ*_(*ω*_1,_*ω*_2_) > 0 and *T*_*ψ*_(*ω*_2,_*ω*_1_) > 0. Thus from Lemma 1, it follows that *α*_1_ < 0 and *α*_2_ < 0, Hence from relation (14) we conclude that ∀*t*, *ω*(*t*) < 0, a contradiction, because according to Corollary 1, ∀t ∈ [0,*I*_max_], *ω*(*t*) ≥ 0.

**Theorem 1.** The function *ω*(*t*) has an extremum if and only if

$$\text{sgn}\left(T_{\psi }(\omega_{1},\omega_{2})*T_{\psi }(\omega_{2},\omega_{1})\right)=1.$$ (24)

In addition, this only extremum, if it exists, is a global minimum at $t^{**}=\frac{I_{\text{max}}}{2\psi }\text{ln}\left(\frac{\alpha_{1}}{\alpha_{2}}\right)$. Also t** ∈ [0,*I*_max_] if and only if

$$\left|\text{ln}\left(\frac{T_{\psi }(\omega_{1},\omega_{2})}{T_{\psi }(\omega_{2},\omega_{1})}\right)\right|\leq \psi .$$ (25)

**Proof.** We first calculate *ω*′(*t*) and *ω*″(*t*) as follows:

$$\omega^{\prime }(t)=\frac{\psi }{I_{{}_{\text{max}}}}\left(-\alpha_{{}_{1}}e^{{}^{\frac{-\psi t}{I_{\text{max}}}}}+\alpha_{{}_{2}}e^{{}^{\frac{\psi t}{I_{\text{max}}}}}\right)$$ (26)

$$\omega^{\prime \prime }(t)={\left(\frac{\psi }{I_{\text{max}}}\right)}^{2}\omega (t)$$ (27)

To find the critical numbers of differentiable function *ω*(*t*), we set its derivative equal to 0. The equation *ω*′(*t*) = 0 implies $t^{**}=\frac{I_{\text{max}}}{2\psi }\text{ln}\left(\frac{\alpha_{1}}{\alpha_{2}}\right)$. Thus we should have $\frac{\alpha_{1}}{\alpha_{2}}>0$ or *α*_1_*α*_2_ > 0. Using Lemma 1 and Corollary 2, it is equivalent to sgn(*T*_*ψ*_(*ω*_1,_*ω*_2_) * *T*_*ψ*_(*ω*_2,_*ω*_1_)) = 1. To use the second derivative test, we evaluate *ω*″(*t*) at this critical number:

$$\omega^{\prime \prime }(t^{**})=2{\left(\frac{\psi }{I_{\text{max}}}\right)}^{2}\sqrt{\alpha_{1}\alpha_{2}}$$

Because of *ω*″(*t*) > 0, *ω*(*t*) has a local minimum at t**, but *α*_1_ > 0 and *α*_2_ > 0 thus

$$\underset{t\to -\infty }{\text{lim}}\begin{array}{c} \omega (t) \end{array}=\underset{t\to +\infty }{\text{lim}}\begin{array}{c} \omega (t) \end{array}=+\infty$$

and so t** is a global minimum of differentiable function *ω*(*t*). The proof of the second part of this Theorem is similar to that of Lemma 2.

**Theorem 2.** If

$$\text{sgn}\left(T_{\psi }(\omega_{1},\omega_{2})\right)=1\begin{array}{ccc} & \text{and} & \end{array}\text{sgn}\left(T_{\psi }(\omega_{2},\omega_{1})\right)=-1,$$ (28)

then *ω*(*t*) is increasing on ℝ and is decreasing on ℝ if

$$\text{sgn}\left(T_{\psi }(\omega_{1},\omega_{2})\right)=-1\begin{array}{ccc} & \text{and} & \end{array}\text{sgn}\left(T_{\psi }(\omega_{2},\omega_{1})\right)=1.$$ (29)

**Proof.** From Lemma 1 and relation (28), we have *α*_1_ < 0 and *α*_2_ > 0, so

$$\alpha_{1}e^{\frac{-\psi t}{I_{\text{max}}}}<0\begin{array}{ccc} & \text{and} & \alpha_{2}e^{\frac{\psi t}{I_{\text{max}}}}>0 \end{array}.$$

Thus $\alpha_{2}e^{\frac{\psi t}{I_{\text{max}}}}>\alpha_{1}e^{\frac{-\psi t}{I_{\text{max}}}}$, this implies

$$\omega^{\prime }(t)=\frac{\psi }{I_{{}_{\text{max}}}}\left(-\alpha_{{}_{1}}e^{{}^{\frac{-\psi t}{I_{\text{max}}}}}+\alpha_{{}_{2}}e^{{}^{\frac{\psi t}{I_{\text{max}}}}}\right)>0$$ (30)

Therefore *ω*(*t*) is increasing on ℝ. The proof of decreasing is similar to increasing.

**Lemma 3.** If *T*_*ψ*_(*ω*_1,_*ω*_2_) = 0 and *ω*_1_ < *ω*_2_ then *ω*(*t*) is increasing. Also If *T*_*ψ*_(*ω*_2,_*ω*_1_) = 0 and *ω*_1_ > *ω*_2_ then *ω*(*t*) is decreasing.

**Proof.** If *T*_*ψ*_(*ω*_1,_*ω*_2_) = 0 then *α*_1_ = 0 and *ω*_2_ − *ω*_1_*e*^*ψ*^ = 0. This implies $\psi =\text{ln}\left(\frac{\omega_{2}}{\omega_{1}}\right)$ and *ψ* > 0 because of *ω*_1_ < *ω*_2_. In this case, we can conclude from Eq (16) that *α*_2_ = *ω*_1_, thus using Eq (14),

$$\omega (t)=\alpha_{2}e^{\frac{\psi t}{I_{\text{max}}}}=\omega_{1}e^{\frac{\psi t}{I_{\text{max}}}}=\omega_{2}e^{\psi \left(\frac{t}{I_{\text{max}}}-1\right)}.$$ (31)

Therefore $\omega^{\prime }(t)=\frac{\omega_{1}\psi }{I_{\text{max}}}e^{\frac{\psi t}{I_{\text{max}}}}>0$ and *ω*(*t*) is increasing. Now suppose *T*_*ψ*_(*ω*_2,_*ω*_1_) = 0 thus *α*_2_ = 0 and *ω*_1_ − *ω*_2_*e*^*ψ*^ = 0. This implies $\psi =\text{ln}\left(\frac{\omega_{1}}{\omega_{2}}\right)$ and *ψ* > 0 because of *ω*_1_ > *ω*_2_. Also *α*_1_ = *ω*_1_ and from Eq (14),

$$\omega (t)=\alpha_{1}e^{\frac{-\psi t}{I_{\text{max}}}}=\omega_{1}e^{\frac{-\psi t}{I_{\text{max}}}}=\omega_{2}e^{\psi \left(1-\frac{t}{I_{\text{max}}}\right)}.$$ (32)

Therefore $\omega^{\prime }(t)=\frac{-\omega_{1}\psi }{I_{\text{max}}}e^{\frac{-\psi t}{I_{\text{max}}}}<0$ and *ω*(*t*) is decreasing.

**Corollary 3.** For all t ∈ [0,*I*_max_], *ω*(*t*) > 0.

**Proof.** By Corollary 1, ∀ t ∈ [0,*I*_max_], *ω*(*t*) ≥ 0. Suppose that ∃t* ∈ [0,*I*_max_], *ω*(t*) = 0. Using Lemma 2, we have sgn(*T*_*ψ*_(*ω*_1,_*ω*_2_) * *T*_*ψ*_(*ω*_2,_*ω*_1_)) = −1. By Theorem 2, *ω*(*t*) is increasing or decreasing. Thus according to relations (17) and (18), ∀t ∈ [0,*I*_max_], *ω*(*t*) ≠ 0, a contradiction. Therefore ∀ t ∈ [0,*I*_max_], *ω*(*t*) > 0.

**Corollary 4.** If *ω*_1_ = *ω*_2_ then *ω*(*t*) takes its global minimum in [0,*I*_max_] at $t^{**}=\frac{I_{\text{max}}}{2}$.

**Proof.** Suppose that *ω*_1_ = *ω*_2_ = Ω. From Eqs (15) and (16), we have *α*_1_ = *e*^*ψ*^*α*_2_, thus using Eq (14), it is concluded that

$$\omega (t)=\frac{\Omega }{1+e^{\psi }}e^{\frac{\psi t}{I_{\text{max}}}}\left(1+e^{\psi (1-\frac{2t}{I_{\text{max}}})}\right).$$ (33)

In this special case, the check functions are as follows:

$$T_{\psi }(\omega_{1},\omega_{2})=T_{\psi }(\omega_{2},\omega_{1})=T_{\psi }(\Omega ,\Omega )=\Omega (1-e^{\psi })<0$$ (34)

By Theorem 1, has a minimum at $t^{**}=\frac{I_{\text{max}}}{2\psi }\text{ln}\left(\frac{\alpha_{1}}{\alpha_{2}}\right)=\frac{I_{\text{max}}}{2\psi }\text{ln}\left(e^{\psi }\right)=\frac{I_{\text{max}}}{2}$.

Thus t** ∈ [0,*I*_max_] and $\omega (t^{**})=\frac{2\Omega }{1+e^{\psi }}e^{\frac{\psi }{2}}$.

**Lemma 4.** As *ψ* approaches 0 from the right, FEIW function approaches linear inertia weight function. If *ω*_1_ > *ω*_2_**,** then this linear function is decreasing, while if *ω*_1_ < *ω*_2_, the function is increasing.

**Proof.** Differentiating *ω*(*t*) with respect to *t*, from Eqs (14)–(16), we get

$$\delta (\psi )=\omega^{\prime }(t)=\frac{\psi }{\left(1-e^{2\psi }\right)I_{{}_{\text{max}}}}\left(-\left(\omega_{2}e^{\psi }-\omega_{1}e^{2\psi }\right)e^{{}^{\frac{-\psi t}{I_{\text{max}}}}}+\left(\omega_{1}-\omega_{2}e^{\psi }\right)e^{{}^{\frac{\psi t}{I_{\text{max}}}}}\right),$$ (35)

so

$$m=\underset{\psi \to 0^{+}}{\text{lim}}\delta (\psi )=\frac{\omega_{2}-\omega_{1}}{I_{{}_{\text{max}}}},$$ (36)

where *m* is the slope of line through (0,*ω*_1_) and (*I*_max_,*ω*_2_). Thus the limit of FEIW function as *ψ* approaches 0 from the right equals $\omega_{\text{lim}}^{\psi }(t)$ as follows:

$$\omega_{\text{lim}}^{\psi }(t)=\frac{\omega_{2}-\omega_{1}}{I_{\text{max}}}\times t+\omega_{1}.$$ (37)

Since *I*_max_ > 0, relation (37) implies $\omega_{\text{lim}}^{\psi }(t)$ is decreasing if *ω*_1_ > *ω*_2_, and is increasing if *ω*_1_ < *ω*_2_.

All of above results are summarized in Table 1.

**Table 1:** Summary of the properties of FEIW function.

Sr. No.	Condition	Conclusion
**1**	$\text{sgn}\left(T_{\psi }(\omega_{1},\omega_{2})*T_{\psi }(\omega_{2},\omega_{1})\right)=-1\begin{array}{c} \;\&\; \end{array}\left|\text{ln}\left(\frac{-T_{\psi }(\omega_{1},\omega_{2})}{T_{\psi }(\omega_{2},\omega_{1})}\right)\right|\leq \psi$	∃! t* ∈ [0,*I*_max_]
**2**	$\text{sgn}\left(T_{\psi }(\omega_{1},\omega_{2})*T_{\psi }(\omega_{2},\omega_{1})\right)=-1\begin{array}{c} \begin{array}{c} \;\&\; \end{array} \end{array}\left|\text{ln}\left(\frac{-T_{\psi }(\omega_{1},\omega_{2})}{T_{\psi }(\omega_{2},\omega_{1})}\right)\right|>\psi$	$\exists !t^{*}\in \mathbb{R}-[0,I_{\text{max}}]$
**3**	$\text{sgn}\left(T_{\psi }(\omega_{1},\omega_{2})*T_{\psi }(\omega_{2},\omega_{1})\right)=1\begin{array}{c} \begin{array}{c} \;\&\; \end{array} \end{array}\left|\text{ln}\left(\frac{T_{\psi }(\omega_{1},\omega_{2})}{T_{\psi }(\omega_{2},\omega_{1})}\right)\right|\leq \psi$	∃! t** ∈ [0,*I*_max_]
**4**	$\text{sgn}\left(T_{\psi }(\omega_{1},\omega_{2})*T_{\psi }(\omega_{2},\omega_{1})\right)=1\begin{array}{c} \begin{array}{c} \;\&\; \end{array} \end{array}\left|\text{ln}\left(\frac{T_{\psi }(\omega_{1},\omega_{2})}{T_{\psi }(\omega_{2},\omega_{1})}\right)\right|>\psi$	$\exists !t^{**}\in \mathbb{R}-[0,I_{\text{max}}]$
**5**	sgn(*T*_*ψ*_(*ω*_1,_*ω*_2_)) = 1 & sgn(*T*_*ψ*_(*ω*_2,_*ω*_1_)) = −1	*ω*(*t*) Increasing on ℝ
**6**	sgn(*T*_*ψ*_(*ω*_1,_*ω*_2_)) = −1 & sgn(*T*_*ψ*_(*ω*_2,_*ω*_1_)) = 1	*ω*(*t*) Decreasing on ℝ

The notations t* and t** represent root of the equation *ω*(*t*) = 0 and minimum of the function *ω*(*t*), respectively.

### 4.3 Flexible exponential inertia weight parameters

The massive experiments indicate the proposed algorithm performance can enhance greatly for most problems when *ω*_1_ ≈ 0, *ω*_2_ ≈ 1, *ψ* ≈ 2.6 for increasing FEIW and *ω*_1_ ≈ 1, *ω*_2_ ≈ 0, *ψ* ≈ 2.6 for decreasing FEIW and *ψ* ≈ 5 for cases *ω*_1_ ≈ *ω*_2_. In this paper, the parameters of different variations of FEIW strategy are selected such that include all the different situations such as increasing (decreasing) functions and functions with a global minimum. Let $G=\left(\frac{1+\sqrt{5}}{2}\right)$. In this strategy, according to Table 1, we experimentally select three values for *ψ* as follows:

$$\psi_{1}=G^{2}≃\text{2}.618\begin{array}{ccc} & , & \end{array}\psi_{2}=\sqrt{G}≃\text{1}.272\begin{array}{ccc} & , & \end{array}\psi_{3}=e^{G}≃\text{5}.043$$ (38)

**Fig 2:**
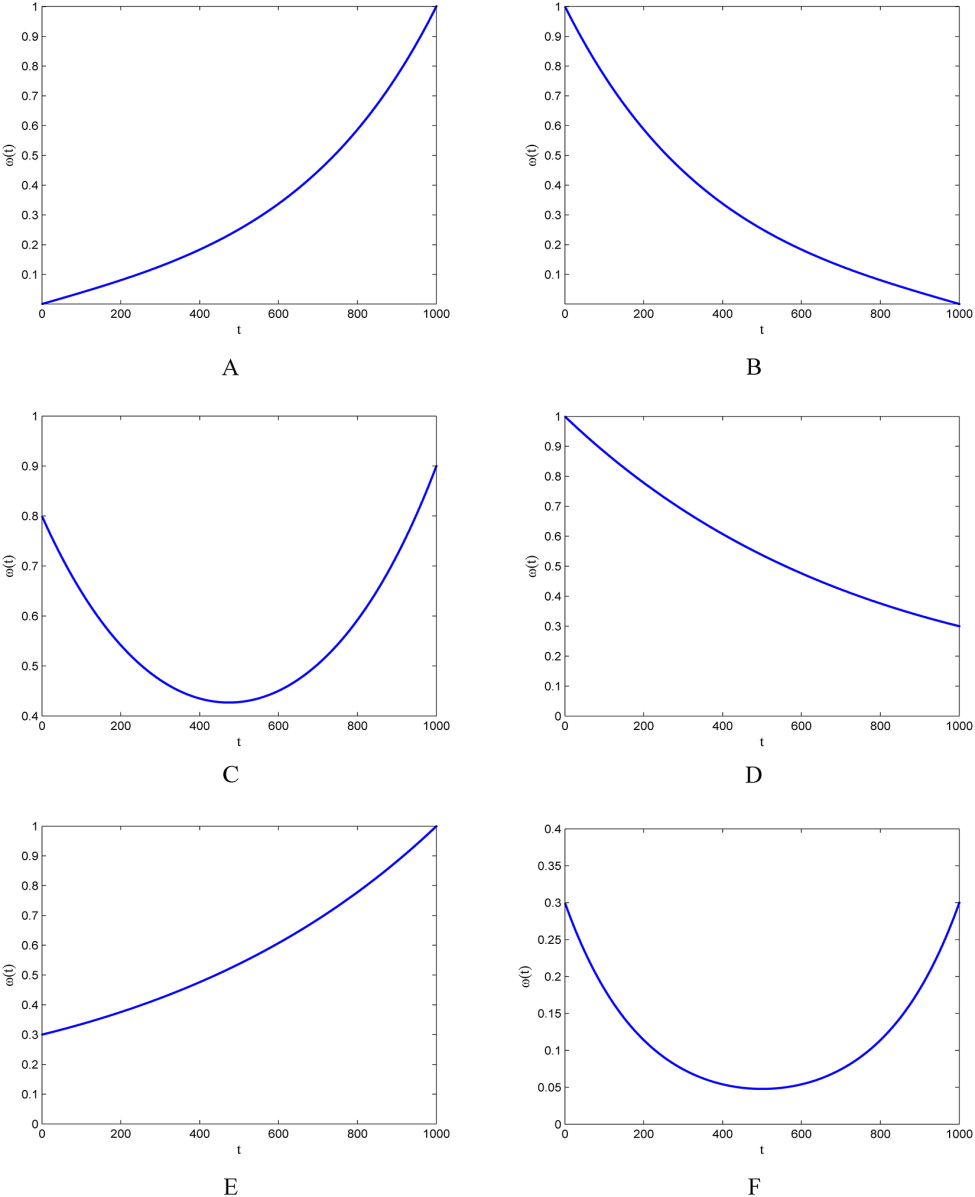
Six variations of Flexible Exponential Inertia Weight (FEIW) strategy. (A) FEIW-1. (B) FEIW-2. (C) FEIW-3. (D) FEIW-4. € FEIW-5. (F) FEIW-6.

Also six pairs of positive numbers are selected for (*ω*_1_,*ω*_2_). These variations of FEIW strategies in Table 2 will be used for comparison with four best IW strategies [27] i.e., CIW, RIW, LDIW and CHIW and four well-known strategies i.e., NEIW, EDIW, GLBIW and AIW. As shown in Fig 2, unlike other inertia weights, the FEIW strategies are either increasing functions or decreasing functions or none.

**Table 2:** The parameters and properties of six variations of FEIW.

Parameters	FEIW-1	FEIW-2	FEIW-3	FEIW-4	FEIW-5	FEIW-6
*ψ*	*ψ*_1_	*ψ*_1_	*ψ*_1_	*ψ*_2_	*ψ*_2_	*ψ*_3_
*ω*_1_	0.001	1.001	0.8	1	0.3	0.3
*ω*_2_	1.001	0.001	0.9	0.3	1	0.3
*α*_1_	-0.072	1.006	0.738	0.994	0.021	0.298
*α*_2_	0.073	-0.005	0.061	0.006	0.279	0.002
*T*_*ψ*_(*ω*_1,_*ω*_2_)	0.987	-13.721	-10.067	-3.268	-0.070	-46.188
*T*_*ψ*_(*ω*_2,_*ω*_1_)	-13.721	0.987	-11.538	-0.070	-3.268	-46.188
$\text{ln}\left(\frac{T_{\psi }(\omega_{1},\omega_{2})}{T_{\psi }(\omega_{2},\omega_{1})}\right)$	—	—	-.0136	3.838	-3.838	0
**Situation**	Increasing on ℝ	Decreasing on ℝ	Minimum at t** ∈ [0,*I*_max_]	Minimum at t** ∉ [0,*I*_max_]	Minimum at t** ∉ [0,*I*_max_]	Minimum at t** ∈ [0,*I*_max_]

## 5 Parameter Settings and Performance Evaluation Criteria

From the standard set of benchmark problems available in the literature, twenty six problems are selected to test efficacy and accuracy of the proposed variants with other existing variants. These problems are of continuous variables and have different degrees of complexity and multimodality. These functions are shown in Tables 3 and 4 along with their range of search space.

**Table 3:** Benchmark functions for simulation.

Function	Name	Search Space	Optimal Value	Reference
*f*_1_	Sphere	[−5.12, 5.12]^*n*^	0	[5, 16, 29]
*f*_2_	Griewank	[−600, 600]^*n*^	0	[5, 16, 29]
*f*_3_	Rosenbrock	[−5, 10]^*n*^	0	[5, 16, 29]
*f*_4_	Rastrigin	[−5.12, 5.12]^*n*^	0	[5, 16]
*f*_5_	Ackley	[−30, 30]^*n*^	0	[5, 16, 29]
*f*_6_	Rotated Hyper-Ellipsoid	[−65.536, 65,536]^*n*^	0	[30]
*f*_7_	Levy	[−10, 10]^*n*^	0	[16]
*f*_8_	Sum squares	[−10, 10]^*n*^	0	[29]
*f*_9_	Zakharov	[−5, 10]^*n*^	0	[29]
*f*_10_	Dixon-Price	[−10, 10]^*n*^	0	[29]
*f*_11_	Schwefel's Problem 2.22	[−10, 10]^*n*^	0	[16, 29, 31]
*f*_12_	Alpine 1	[−10, 10]^*n*^	0	[29]
*f*_13_	Mishra 7	[−10, 10]^*n*^	0	[29]
*f*_14_	Bent-Cigar	[−100, 100]^*n*^	0	[32]
*f*_15_	Noncontinuous Rastrigin	[−5.12, 5.12]^*n*^	0	[5, 16]
*f*_16_	Trigonometric 2	[−500, 500]^*n*^	1	[29]
*f*_17_	Generalized Penalized-1	[−50, 50]^*n*^	0	[31]
*f*_18_	Generalized Penalized-2	[−50, 50]^*n*^	0	[31]
*f*_19_	Weierstrass	[−0.5, 0.5]^*n*^	0	[5]
*f*_20_	Shifted Rotated Weierstrass	[−0.5, 0.5]^*n*^	90	[33]
*f*_21_	Michalewicz	[0, *π*]^10^	-9.66015	[30]
*f*_22_	Quintic	[−10, 10]^*n*^	0	[29]
*f*_23_	Pinter	[−10, 10]^*n*^	0	[29]
*f*_24_	Pathological	[−100, 100]^*n*^	0	[29]
*f*_25_	Salomon	[−100, 100]^*n*^	0	[29]
*f*_26_	Mishra 11	[−10, 10]^*n*^	0	[29]

**Table 4:** Benchmark functions formula.

No.	Objective Functions
**1**	$f_{1}(x)=\sum_{i=1}^{D}x_{i}{}^{2}$
**2**	$f_{2}(x)=\frac{1}{4000}\sum_{i=1}^{D}x_{i}{}^{2}-\prod_{i=1}^{D}\text{cos}\left(\frac{x_{i}}{\sqrt{i}}\right)+1$
**3**	$f_{3}(x)=\sum_{i=1}^{D-1}\left[100{\left(x_{i+1}-x_{i}{}^{2}\right)}^{2}+{\left(x_{i}-1\right)}^{2}\right]$
**4**	$f_{4}(x)=10D+\sum_{i=1}^{D}\left[x_{i}{}^{2}-10\text{cos}(2\pi x_{i})\right]$
**5**	$f_{5}(x)=-20\text{exp}\left(-0.2\sqrt{\frac{1}{D}\sum_{i=1}^{D}x_{i}{}^{2}}\right)-\text{exp}\left(\frac{1}{D}\sum_{i=1}^{D}\text{cos}(2\pi x_{i})\right)+20+e$
**6**	$f_{6}(x)=\sum_{i=1}^{D}\sum_{j=1}^{i}x_{j}{}^{2}$
**7**	$f_{7}(x)={\text{sin}}^{2}(\pi y_{1})+\sum_{i=1}^{D-1}{\left(y_{i}-1\right)}^{2}\left[1+10{\text{sin}}^{2}(\pi y_{i}+1)\right]+{\left(y_{D}-1\right)}^{2}\left[1+{\text{sin}}^{2}(2\pi y_{D})\right],\;y_{i}=1+\frac{x_{i}-1}{4},\;i=1,\ldots ,D$
**8**	$f_{8}(x)=\sum_{i=1}^{D}ix_{i}{}^{2}$
**9**	$f_{9}(x)=\sum_{i=1}^{D}x_{i}{}^{2}+{\left(\sum_{i=1}^{D}0.5ix_{i}\right)}^{2}+{\left(\sum_{i=1}^{D}0.5ix_{i}\right)}^{4}$
**10**	$f_{10}(x)={\left(x_{1}-1\right)}^{2}+\sum_{i=2}^{D}i{\left(2x_{i}{}^{2}-x_{i-1}\right)}^{2}$
**11**	$f_{11}(x)=\sum_{i=1}^{D}|x_{i}\left|+\prod_{i=1}^{D}|x_{i}\right|$
**12**	$f_{12}(x)=\sum_{i=1}^{D}\left|x_{i}\text{sin}(x_{i})+0.1x_{i}\right|$
**13**	$f_{13}(x)={\left(\prod_{i=1}^{D}x_{i}-D!\right)}^{2}$
**14**	$f_{14}(x)=x_{1}{}^{2}+10^{6}\sum_{i=2}^{D}x_{i}{}^{2}$
**15**	$f_{15}(x)=\sum_{i=1}^{D}\left(y_{i}^{2}-10\text{cos}\left(2\pi y_{i}\right)+10\right),\;y_{i}=\{\begin{array}{ll} x_{i}, & \left|x_{i}\right|<\frac{1}{2}\\ \frac{\text{round}(2x_{i})}{2}, & \left|x_{i}\right|\geq \frac{1}{2} \end{array}$
**16**	$f_{16}(x)=1+\sum_{i=1}^{D}8{\text{sin}}^{2}\left[7{\left(x_{i}-0.9\right)}^{2}\right]+6{\text{sin}}^{2}\left[14{\left(x_{i}-0.9\right)}^{2}\right]+{\left(x_{i}-0.9\right)}^{2}$
**17**	$f_{17}(x)=\frac{\pi }{D}\left\{10{\text{sin}}^{2}(\pi y_{1})+\sum_{i=1}^{D-1}{\left(y_{i}-1\right)}^{2}\left[1+10{\text{sin}}^{2}(\pi y_{i+1})\right]+{\left(y_{D}-1\right)}^{2}\right\}+\sum_{i=1}^{D}\text{u}(x_{i},10,100,4),\;y_{i}=1+\frac{1}{4}(x_{i}+1),\text{ u}(x_{i},a,k,m)=\{\begin{array}{ll} k{\left(x_{i}-a\right)}^{m}, & x_{i}>a\\ 0, & -a\leq x_{i}\leq a\\ k{\left(-x_{i}-a\right)}^{m}, & x_{i}<-a \end{array}$
**18**	$f_{18}(x)=\frac{1}{10}\left\{{\text{sin}}^{2}(3\pi x_{1})+\sum_{i=1}^{D-1}{\left(x_{i}-1\right)}^{2}\left[1+{\text{sin}}^{2}(3\pi x_{i+1})\right]+{\left(x_{D}-1\right)}^{2}[1+{\text{sin}}^{2}(2\pi x_{D})]\right\}+\sum_{i=1}^{D}\text{u}(x_{i},5,100,4)$
**19**	$f_{19}(x)=\sum_{i=1}^{D}\left(\sum_{k=0}^{k_{max}}\left[a^{k}\text{cos}\left(2\pi b^{k}(x_{i}+0.5)\right)\right]\right)-D\sum_{k=0}^{k_{max}}\left[a^{k}\text{cos}\left(2\pi b^{k}\cdot 0.5\right)\right],\;a=0.5\;b=3,\;k_{max}=20$
**20**	$f_{20}(x)=\sum_{i=1}^{D}\left(\sum_{k=0}^{k_{max}}\left[a^{k}\text{cos}\left(2\pi b^{k}(z_{i}+0.5)\right)\right]\right)-D\sum_{k=0}^{k_{max}}\left[a^{k}\text{cos}\left(2\pi b^{k}\cdot 0.5\right)\right]+f_{\text{bias}},$ $z=M*(x-o),\;a=0.5,\;b=3,\;k_{max}=20,\;f_{\text{bias}}=90,$ M:linear transformation matrix, condition number=5,$o=[o_{1},o_{2},\ldots ,o_{D}]:\;\text{the shifted global optimum}$
**21**	$f_{21}(x)=-\sum_{i=1}^{D}\text{sin}\left(x_{i}\right){\left[\text{sin}\left(\frac{ix_{i}{}^{2}}{\pi }\right)\right]}^{2m},m=10$
**22**	$f_{22}(x)=\sum_{i=1}^{D}\left|x_{i}{}^{5}-3x_{i}{}^{4}+4x_{i}{}^{3}+2x_{i}{}^{2}-10x_{i}-4\right|$
**23**	$f_{23}(x)=\sum_{i=1}^{D}\left[ix_{i}{}^{2}+20i{\text{sin}}^{2}(A)+\text{ilo}{\text{g}}_{10}\left(1+iB^{2}\right)\right],$ $A=x_{i-1}\text{sin}x_{i}+\text{sin}x_{i+1},B=x_{i-1}{}^{2}-2x_{i}+3x_{i+1}-\text{cos}x_{i}+1,\text{where }x_{0}=x_{D}\text{ and }x_{D+1}=x_{1}$
**24**	$f_{24}(x)=\sum_{i=1}^{D-1}\left(0.5+\frac{{\text{sin}}^{2}\sqrt{100x_{i}{}^{2}+x_{i+1}{}^{2}}-0.5}{1+0.001{\left(x_{i}{}^{2}-2x_{i}x_{i+1}+x_{i+1}{}^{2}\right)}^{2}}\right)$
**25**	$f_{25}(x)=1-\text{cos}\left(2\pi \sqrt{\sum_{i=1}^{D}x_{i}{}^{2}}\right)+0.1\sqrt{\sum_{i=1}^{D}x_{i}{}^{2}}$
**26**	$f_{26}(x)={\left[\frac{1}{D}\sum_{i=1}^{D}|x_{i}|+{\left(\prod_{i=1}^{D}|x_{i}|\right)}^{\frac{1}{D}}\right]}^{2}$

### 5.1 Parameter settings

For implementing these fourteen strategies in PSO, a code has been developed in MATLAB^®^ 2014. For a fair comparison, all the fourteen variants are run with the same parameter setting and on same computing environment. Each PSO variant is run 100 times with random initial population.

➢ Swarm size: *M* = 5 × *D*.
➢ Problem size: *D* = 10, 50.
➢ Acceleration coefficients: *c*_1_ = *c*_2_ = 2.
➢ Maximum velocity: *v*_max_ = 0.1 × (*x*_max_ − *x*_min_)
➢ Maximum number of iterations allowed: *I*_max_ = 500, 1000.

### 5.2 Performance evaluation criteria (PEC)

According to the “no free lunch theorem” [34], one optimization algorithm cannot offer better performance than all the others on every aspect or on every kind of problem. Thus the efficiency and accuracy of all algorithms is tested against a set of well-known standard benchmark unimodal and multimodal functions given in Tables 3 and 4. Also we use of different evaluation criteria to obtain valid results. A run in which the algorithm finds a solution satisfying |*f_out_* − *f*_min_| < *ε*, where *f_out_* is the best solution found when the algorithm terminates and *f*_min_ is the known global minimum of the problem, is considered to be successful. In this case, *ε* is error of the algorithm. In order to evaluate the performance of different IW strategies, we need to define different terms for termination of the PSO algorithm, so the termination criterion for all considered PSO variants is one of the following conditions:

➢ Condition 1: achieving to *I*_max_.
➢ Condition 2: achieving to *I*_max_ or when the known optimum is within 1 –*ε* of accuracy, whichever occurs earlier.

For each method and problem the following are recorded:

1. Success rate (*SR*) is number of successful runs (*S*_*run*_) per total number of runs (*T*_*run*_)
  $$SR=\frac{S_{run}}{T_{run}}\times 100$$ (39)
2. Average number of iterations of successful runs (*ANS*).
3. Minimum number of iterations of successful runs (*MNS*).
4. Average error (*AE*),
  $$AE=\frac{\underset{T_{run}}{\sum }\left|f_{out}-f_{\text{min}}\right|}{T_{run}}.$$ (40)
5. Minimum error (*ME*) over 100 runs.
6. Standard deviation (*STD*) of error over 100 runs.

## 6 Results, Analysis and Discussions

### 6.1 Numerical results

In this subsection, a comprehensive comparative study of IW for fourteen strategies is carried out. The computational results for all the considered set of benchmark functions using all the PSO variants, comprises results for the all mentioned performance evaluation criteria (PEC) over 100 runs. The numerical results are shown in Tables 5–14.

**Table 5:** Comparison of success rate, average and minimum number of iterations of successful runs for considered PSO variants with condition 2, *I*_max_ = 1000, *D* = 10, *ε* = 10^−1^ for *f*_2_, *f*_3_, *f*_4_, *f*_10_ functions and *ε* = 10^−10^ for others (*υ* > *I*_max_).

IW	PEC	*f*_1_	*f*_2_	*f*_3_	*f*_4_	*f*_5_	*f*_6_	*f*_7_	*f*_8_	*f*_9_	*f*_10_
**CIW**	*SR*	100	42	2	0	0	88	100	100	0	3
	*ANS*	659	578	791	*υ*	*υ*	900	696	769	*υ*	755
	*MNS*	537	232	612	*υ*	*υ*	719	557	612	*υ*	250
**RIW**	*SR*	0	6	2	0	0	0	0	0	0	7
	*ANS*	*υ*	769	684	*υ*	*υ*	*υ*	*υ*	*υ*	*υ*	449
	*MNS*	*υ*	647	587	*υ*	*υ*	*υ*	*υ*	*υ*	*υ*	265
**LDIW**	*SR*	100	78	1	0	100	100	100	100	100	6
	*ANS*	667	695	878	*υ*	882	729	668	694	875	853
	*MNS*	630	448	878	*υ*	847	697	632	651	816	455
**CHIW**	*SR*	100	83	2	0	100	100	100	100	100	6
	*ANS*	420	495	644	*υ*	639	484	428	448	649	626
	*MNS*	367	242	376	*υ*	590	423	388	394	569	205
**FEIW-1**	*SR*	100	73	25	0	96	100	97	100	100	9
	*ANS*	57	195	216	*υ*	145	76	65	62	317	295
	*MNS*	41	18	24	*υ*	105	55	47	46	260	18
**FEIW-2**	*SR*	100	77	3	0	100	100	100	100	100	6
	*ANS*	319	382	537	*υ*	442	356	322	334	459	446
	*MNS*	299	240	316	*υ*	420	339	305	312	426	213
**FEIW-3**	*SR*	100	87	4	0	100	100	99	100	100	3
	*ANS*	274	337	346	*υ*	445	320	280	296	450	441
	*MNS*	251	167	186	*υ*	418	295	251	271	401	154
**FEIW-4**	*SR*	100	81	1	0	100	100	100	100	100	2
	*ANS*	522	583	560	*υ*	706	573	526	545	707	702
	*MNS*	491	400	560	*υ*	682	544	497	515	652	418
**FEIW-5**	*SR*	100	80	11	0	100	100	96	100	100	9
	*ANS*	95	125	136	*υ*	224	121	102	106	276	255
	*MNS*	78	36	53	*υ*	174	102	83	83	223	24
**FEIW-6**	*SR*	100	77	9	0	99	100	99	100	100	12
	*ANS*	77	163	431	*υ*	158	98	84	86	257	234
	*MNS*	66	36	47	*υ*	142	87	66	67	204	38

**Table 6:** Comparison of success rate, average and minimum number of iterations of successful runs for considered PSO variants with condition 2, *I*_max_ = 1000, *D* = 10, *ε* = 5 for *f*_15_ and *f*_20_ functions, *ε* = 10^−1^ for *f*_19_, *f*_21_, *f*_24_, *f*_25_ functions and *ε* = 10^−10^ for others (*υ* > *I*_max_).

IW	PEC	*f*_11_	*f*_12_	*f*_13_	*f*_14_	*f*_15_	*f*_16_	*f*_17_	*f*_18_	*f*_19_	*f*_20_	*f*_21_	*f*_22_	*f*_23_	*f*_24_	*f*_25_	*f*_26_
**GLBIW**	*SR*	0	0	0	0	10	0	0	0	90	25	0	0	0	84	68	2
	*ANS*	*υ*	*υ*	*υ*	*υ*	420	*υ*	*υ*	*υ*	131	67	*υ*	*υ*	*υ*	331	373	460
	*MNS*	*υ*	*υ*	*υ*	*υ*	303	*υ*	*υ*	*υ*	96	10	*υ*	*υ*	*υ*	74	206	270
**AIW**	*SR*	0	0	1	0	50	0	0	0	95	90	0	0	0	47	4	64
	*ANS*	*υ*	*υ*	961	*υ*	676	*υ*	*υ*	*υ*	460	119	*υ*	*υ*	*υ*	565	943	666
	*MNS*	*υ*	*υ*	961	*υ*	430	*υ*	*υ*	*υ*	334	18	*υ*	*υ*	*υ*	135	916	274
**NEIW**	*SR*	100	100	72	100	10	98	100	100	100	90	0	100	74	93	70	100
	*ANS*	499	489	392	482	290	529	378	390	281	167	*υ*	537	424	390	401	247
	*MNS*	481	470	321	455	274	445	358	352	266	76	*υ*	501	399	259	274	220
**EDIW**	*SR*	100	100	66	100	10	96	100	100	100	95	0	100	83	96	66	100
	*ANS*	415	410	302	396	350	454	279	300	182	101	*υ*	461	339	285	314	140
	*MNS*	398	367	223	375	264	355	252	272	166	24	*υ*	431	312	136	184	108
**FEIW-1**	*SR*	100	100	82	100	53	98	94	100	99	95	6	100	85	97	71	100
	*ANS*	119	122	375	108	10	387	70	89	53	265	27	140	104	203	302	27
	*MNS*	101	96	47	91	10	148	49	57	27	19	23	114	80	32	79	19
**FEIW-2**	*SR*	100	100	58	100	7	94	100	100	95	95	0	100	87	96	68	100
	*ANS*	426	419	363	412	319	451	328	343	243	138	*υ*	454	365	328	331	202
	*MNS*	411	390	280	391	195	379	315	323	225	82	*υ*	432	344	218	224	39
**FEIW-3**	*SR*	100	100	62	100	57	98	100	100	100	90	0	100	76	96	74	100
	*ANS*	416	410	304	395	626	451	284	302	182	171	*υ*	454	341	285	320	139
	*MNS*	390	372	239	368	88	370	266	281	167	36	*υ*	422	308	99	144	78
**FEIW-4**	*SR*	100	100	80	100	20	98	100	100	95	100	0	100	81	100	73	100
	*ANS*	674	668	528	658	461	699	533	548	404	210	*υ*	720	592	497	535	352
	*MNS*	659	635	465	630	354	605	502	527	368	30	*υ*	684	561	300	381	309
**FEIW-5**	*SR*	100	100	52	100	54	84	94	100	100	45	5	100	66	79	64	100
	*ANS*	195	190	242	179	726	249	103	114	53	48	52	228	144	151	263	38
	*MNS*	177	162	101	159	20	177	81	97	42	19	36	197	117	43	91	26
**FEIW-6**	*SR*	100	100	14	100	14	64	100	100	100	45	5	100	66	83	65	100
	*ANS*	143	141	480	134	109	252	89	94	54	115	28	162	122	210	305	35
	*MNS*	131	129	109	118	34	125	74	79	41	23	23	143	90	51	74	21

**Table 7:** Comparison of average, minimum and standard deviation of error for considered PSO variants with condition 1, *I*_max_ = 1000 and *D* = 10.

IW	PEC	*f*_1_	*f*_2_	*f*_3_	*f*_4_	*f*_5_
**CIW**	*AE*	4.438e-14	7.679e-02	3.256e+00	6.169e+00	9.191e-07
	*ME*	1.978e-16	3.021e-02	4.073e-01	1.990e+00	7.529e-08
	*STD*	6.737e-14	2.874e-02	2.291e+00	2.959e+00	1.193e-06
**RIW**	*AE*	1.071e-06	3.126e-01	5.975e+00	4.977e+00	8.072e-03
	*ME*	1.271e-08	2.121e-01	5.894e-01	1.990e+00	5.490e-04
	*STD*	1.381e-06	8.337e-02	1.985e+00	1.960e+00	5.167e-03
**LDIW**	*AE*	4.935e-06	4.101e-01	6.413e+00	5.975e+00	1.504e-02
	*ME*	4.273e-07	4.477e-02	3.431e+00	1.764e+00	4.980e-03
	*STD*	3.605e-06	2.103e-01	1.075e+00	3.501e+00	1.027e-02
**CHIW**	*AE*	3.438e-06	2.779e-01	6.646e+00	5.578e+00	1.338e-02
	*ME*	4.884e-07	7.319e-02	5.369e+00	2.988e+00	5.368e-03
	*STD*	3.100e-06	1.339e-01	5.057e-01	1.707e+00	7.816e-03
**FEIW-1**	*AE*	1.732e-159	9.351e-02	1.432e+00	8.457e+00	4.441e-15
	*ME*	6.886e-171	4.180e-02	6.209e-02	9.950e-01	4.441e-15
	*STD*	5.476e-159	3.202e-02	1.548e+00	7.035e-01	1.00e-310
**FEIW-2**	*AE*	1.412e-30	7.012e-02	4.188e+00	5.423e+00	8.882e-15
	*ME*	7.348e-34	2.464e-02	2.408e+00	9.950e-01	4.441e-15
	*STD*	3.585e-30	2.462e-02	1.026e+00	2.767e+00	4.873e-15
**FEIW-3**	*AE*	3.088e-38	7.480e-02	3.225e+00	7.373e+00	4.796e-15
	*ME*	7.679e-42	1.723e-02	5.636e-02	2.985e+00	4.441e-15
	*STD*	1.337e-37	3.129e-02	1.131e+00	3.131e+00	1.071e-15
**FEIW-4**	*AE*	9.719e-07	2.938e-01	5.890e+00	5.399e+00	6.693e-03
	*ME*	9.825e-08	5.367e-02	7.974e-01	1.990e+00	1.134e-03
	*STD*	9.088e-07	1.484e-01	1.805e+00	2.563e+00	6.805e-03
**FEIW-5**	*AE*	1.851e-91	8.561e-02	1.769e+00	1.094e+01	4.441e-15
	*ME*	1.276e-97	6.896e-02	8.838e-02	1.094e+01	4.441e-15
	*STD*	5.760e-91	1.868e-02	1.091e+00	1.00e-310	1.00e-310
**FEIW-6**	*AE*	1.462e-151	7.832e-02	1.807e+00	7.761e+00	4.441e-15
	*ME*	3.925e-159	3.937e-02	2.629e-02	3.980e+00	4.441e-15
	*STD*	4.455e-151	3.633e-02	2.474e+00	2.727e+00	1.00e-310

**Table 8:** Comparison of average, minimum and standard deviation of error for considered PSO variants with condition 1, *I*_max_ = 1000 and *D* = 10.

IW	PEC	*f*_6_	*f*_7_	*f*_8_	*f*_9_	*f*_10_
**CIW**	*AE*	1.070e-11	1.170e-12	6.570e-13	1.580e-06	6.222e-01
	*ME*	1.530e-13	2.870e-16	9.630e-15	7.500e-08	1.650e-07
	*STD*	1.850e-11	4.330e-12	6.990e-13	2.440e-06	1.721e-01
**RIW**	*AE*	1.250e-03	1.150e-01	7.750e-05	1.630e-03	6.241e-01
	*ME*	3.800e-06	3.730e-08	3.940e-06	2.610e-04	2.750e-02
	*STD*	1.600e-03	4.460e-01	1.550e-04	1.210e-03	1.651e-01
**LDIW**	*AE*	2.350e-01	5.830e-04	6.740e-03	1.670e-02	7.003e-01
	*ME*	1.730e-02	7.660e-05	6.430e-04	5.530e-03	6.670e-01
	*STD*	2.290e-01	6.390e-04	8.150e-03	6.940e-03	9.322e-02
**CHIW**	*AE*	2.450e-14	6.870e-17	8.550e-16	8.160e-08	6.667e-01
	*ME*	1.600e-18	6.670e-20	9.470e-19	3.810e-10	6.670e-01
	*STD*	3.700e-14	1.120e-16	1.930e-15	1.610e-07	9.520e-12
**FEIW-1**	*AE*	9.980e-155	3.030e-02	1.470e-155	1.800e-30	8.889e-02
	*ME*	9.140e-168	9.140e-168	2.910e-172	7.510e-35	1.550e-34
	*STD*	2.540e-154	1.170e-01	5.680e-155	3.060e-30	2.346e-01
**FEIW-2**	*AE*	2.140e-28	7.060e-31	5.510e-29	1.710e-14	5.778e-01
	*ME*	1.830e-30	1.500e-32	1.760e-32	2.970e-17	2.080e-16
	*STD*	4.880e-28	1.100e-30	1.240e-28	1.900e-14	2.346e-01
**FEIW-3**	*AE*	2.170e-35	1.500e-32	3.420e-37	6.470e-18	5.778e-01
	*ME*	1.250e-38	1.500e-32	7.380e-41	5.480e-20	3.140e-23
	*STD*	5.730e-35	2.830e-48	9.890e-37	1.680e-17	2.346e-01
**FEIW-4**	*AE*	4.780e-04	1.330e-06	1.360e-05	1.155e-03	6.669e-01
	*ME*	2.740e-05	1.490e-07	7.750e-07	2.600e-04	6.667e-01
	*STD*	4.940e-04	1.950e-06	2.180e-05	7.380e-04	7.800e-04
**FEIW-5**	*AE*	1.790e-87	1.500e-32	2.210e-90	1.690e-35	8.889e-02
	*ME*	1.430e-95	1.500e-32	2.080e-95	5.650e-41	5.650e-41
	*STD*	6.780e-87	2.830e-48	6.620e-90	6.440e-35	2.346e-01
**FEIW-6**	*AE*	9.960e-147	1.500e-32	4.120e-148	1.690e-37	4.440e-02
	*ME*	3.990e-161	1.500e-32	1.980e-161	1.310e-44	3.950e-43
	*STD*	2.670e-146	2.830e-48	1.530e-147	3.430e-37	1.721e-01

**Table 9:** Comparison of average, minimum and standard deviation of error for considered PSO variants with condition 1, *I*_max_ = 1000 and *D* = 10.

IW	PEC	*f*_11_	*f*_12_	*f*_13_	*f*_14_	*f*_15_	*f*_16_	*f*_17_	*f*_18_
**GLBIW**	*AE*	1.29e-01	1.59e-01	7.87e+04	9.24e+06	6.73e+00	2.70e+02	3.24e-01	4.93e-01
	*ME*	6.69e-02	3.60e-02	1.20e+02	4.57e+06	2.00e+00	7.20e+01	1.60e-01	1.21e-01
	*STD*	5.26e-02	8.86e-02	2.93e+05	3.39e+06	2.45e+00	1.06e+02	9.58e-02	1.91e-01
**AIW**	*AE*	3.98e-03	2.11e-03	6.84e+03	7.03e+03	4.30e+00	2.23e+01	2.15e-04	3.16e-03
	*ME*	2.29e-04	2.05e-04	2.30e-03	1.17e+02	2.02e+00	6.34e+00	3.83e-06	1.01e-04
	*STD*	3.13e-03	1.40e-03	1.61e+04	7.52e+03	1.11e+00	8.42e+00	2.18e-04	3.95e-03
**NEIW**	*AE*	1.73e-32	4.96e-16	8.32e-02	1.90e-50	6.30e+00	1.00e-300	4.71e-32	1.35e-32
	*ME*	5.76e-35	8.13e-60	1.00e-300	1.98e-57	4.00e+00	1.00e-300	4.71e-32	1.35e-32
	*STD*	3.16e-32	7.76e-16	3.54e-01	6.15e-50	1.42e+00	1.00e-300	1.67e-47	5.57e-48
**EDIW**	*AE*	1.03e-34	6.12e-16	1.43e-01	3.39e-53	6.20e+00	1.00e-300	4.71e-32	1.35e-32
	*ME*	2.74e-38	3.50e-52	1.00e-300	4.30e-60	3.00e+00	1.00e-300	4.71e-32	1.35e-32
	*STD*	2.37e-34	9.36e-16	7.82e-01	1.82e-52	2.06e+00	1.00e-300	1.67e-47	5.57e-48
**FEIW-1**	*AE*	2.99e-53	3.48e-16	1.22e-04	9.20e-95	5.09e+00	1.00e-300	4.71e-32	1.35e-32
	*ME*	1.12e-61	6.72e-79	1.00e-300	1.78e-106	2.00e+00	1.00e-300	4.71e-32	1.35e-32
	*STD*	1.34e-52	7.89e-16	4.63e-04	2.78e-94	2.47e+00	1.34e+00	7.89e-02	2.01e-03
**FEIW-2**	*AE*	3.67e-54	5.53e-16	9.40e-01	5.71e-95	6.13e+00	2.99e-02	4.71e-32	1.35e-32
	*ME*	3.12e-60	7.17e-122	1.00e-300	2.78e-108	4.00e+00	1.00e-300	4.71e-32	1.35e-32
	*STD*	1.62e-53	9.11e-16	4.44e+00	2.44e-94	1.22e+00	1.64e-01	1.67e-47	5.57e-48
**FEIW-3**	*AE*	1.75e-25	8.96e-16	1.19e+00	6.09e-38	3.68e+00	4.67e-12	4.71e-32	1.35e-32
	*ME*	9.02e-28	5.26e-38	1.00e-300	1.97e-42	1.05e-04	1.00e-300	4.71e-32	1.35e-32
	*STD*	2.97e-25	1.82e-15	6.48e+00	2.40e-37	1.77e+00	2.56e-11	1.67e-47	5.57e-48
**FEIW-4**	*AE*	2.88e-26	4.07e-16	3.47e+01	4.78e-40	5.70e+00	5.98e-02	4.71e-32	1.35e-32
	*ME*	8.39e-28	9.40e-36	1.00e-300	1.64e-44	3.00e+00	1.00e-300	4.71e-32	1.35e-32
	*STD*	8.31e-26	7.29e-16	1.90e+02	1.52e-39	1.93e+00	2.28e-01	1.67e-47	5.57e-48
**FEIW-5**	*AE*	2.77e-26	7.83e-16	2.47e-01	2.20e-39	4.58e+00	1.79e-01	4.71e-32	1.40e-32
	*ME*	5.80e-29	1.26e-38	1.00e-300	3.79e-46	1.25e+00	1.00e-300	4.71e-32	1.35e-32
	*STD*	7.48e-26	9.83e-16	1.21e+00	9.20e-39	1.55e+00	6.93e-01	1.67e-47	2.70e-33
**FEIW-6**	*AE*	3.27e-55	4.87e-16	3.86e-03	4.72e-144	8.00e+00	1.50e-01	4.71e-32	3.66e-04
	*ME*	1.69e-88	3.48e-148	4.95e-11	2.70e-158	4.00e+00	1.00e-300	4.71e-32	1.35e-32
	*STD*	1.79e-54	4.91e-16	1.82e-02	2.24e-143	3.05e+00	3.79e-01	1.67e-47	2.01e-03

**Table 10:** Comparison of average, minimum and standard deviation of error for considered PSO variants with condition 1, *I*_max_ = 1000 and *D* = 10.

IW	PEC	*f*_19_	*f*_20_	*f*_21_	*f*_22_	*f*_23_	*f*_24_	*f*_25_	*f*_26_
**GLBIW**	*AE*	5.86e-02	6.01e+00	1.06e+00	1.62e+00	2.02e+01	9.85e-02	1.20e-01	3.18e-05
	*ME*	3.50e-03	2.45e+00	1.04e-01	7.79e-01	1.95e+00	3.46e-02	9.99e-02	3.16e-30
	*STD*	4.48e-02	1.35e+00	6.53e-01	5.70e-01	1.87e+01	8.02e-02	4.07e-02	2.13e-05
**AIW**	*AE*	2.35e-02	3.39e+00	5.66e-01	1.03e-01	1.75e+01	1.05e-01	1.97e-01	2.62e-10
	*ME*	4.88e-03	6.94e-01	8.65e-02	1.61e-02	6.85e-03	3.40e-02	9.99e-02	3.96e-15
	*STD*	1.50e-02	1.47e+00	3.15e-01	1.16e-01	4.57e+01	3.78e-02	4.88e-02	6.26e-10
**NEIW**	*AE*	1.00e-01	1.98e+00	5.74e-01	1.00e-300	1.06e+01	4.44e-02	1.37e-01	1.00e-300
	*ME*	1.00e-300	5.02e-01	1.44e-01	1.00e-300	3.19e-61	2.35e-03	9.99e-02	1.00e-300
	*STD*	3.81e-01	1.31e+00	2.95e-01	1.00e-300	1.96e+01	7.48e-02	4.90e-02	1.00e-300
**EDIW**	*AE*	1.00e-300	2.56e+00	6.82e-01	1.18e-16	1.08e+01	1.88e-02	1.20e-01	1.00e-300
	*ME*	1.00e-300	1.00e+00	2.79e-01	1.00e-300	1.12e-64	2.37e-03	9.99e-02	1.00e-300
	*STD*	1.00e-300	1.07e+00	2.87e-01	6.49e-16	2.26e+01	1.57e-02	4.07e-02	1.00e-300
**FEIW-1**	*AE*	1.00e-300	1.28e+00	4.89e-01	2.98e-17	7.42e+00	1.40e-02	1.10e-01	1.00e-300
	*ME*	1.00e-300	4.45e-01	8.24e-02	1.00e-300	3.73e-105	1.30e-03	9.99e-02	1.00e-300
	*STD*	3.61e-01	1.85e+00	2.67e-01	1.61e-13	6.38e+01	7.70e-02	4.98e-02	1.00e-300
**FEIW-2**	*AE*	1.00e-300	2.35e+00	6.45e-01	1.33e-16	1.21e+01	3.41e-02	1.23e-01	1.00e-300
	*ME*	1.00e-300	7.05e-01	6.69e-02	1.00e-300	7.95e-109	3.09e-04	9.99e-02	1.00e-300
	*STD*	1.00e-300	1.42e+00	3.17e-01	6.51e-16	2.04e+01	6.92e-02	4.30e-02	1.00e-300
**FEIW-3**	*AE*	5.00e-02	2.68e+00	5.14e-01	1.00e-300	1.15e+01	4.31e-02	1.23e-01	1.00e-300
	*ME*	1.00e-300	5.89e-01	1.17e-01	1.00e-300	9.57e-47	2.09e-03	9.99e-02	1.00e-300
	*STD*	2.74e-01	1.37e+00	2.75e-01	1.00e-300	2.48e+01	5.28e-02	4.30e-02	1.00e-300
**FEIW-4**	*AE*	1.00e-300	2.23e+00	5.17e-01	1.48e-17	1.52e+00	2.86e-02	1.17e-01	1.00e-300
	*ME*	1.00e-300	4.95e-01	4.11e-02	1.00e-300	2.54e-52	9.71e-04	9.99e-02	1.00e-300
	*STD*	1.00e-300	1.04e+00	3.21e-01	8.11e-17	8.33e+00	3.41e-02	3.79e-02	1.00e-300
**FEIW-5**	*AE*	8.75e-03	6.34e+00	6.02e-01	1.00e-300	1.84e+01	9.17e-02	1.48e-01	1.00e-300
	*ME*	1.00e-300	2.11e+00	8.59e-02	1.00e-300	2.99e-48	2.72e-03	9.99e-02	1.00e-300
	*STD*	4.79e-02	1.64e+00	3.37e-01	1.00e-300	4.23e+01	1.10e-01	5.00e-02	1.00e-300
**FEIW-6**	*AE*	5.25e-04	5.90e+00	6.75e-01	1.48e-16	2.20e+01	8.38e-02	1.30e-01	1.00e-300
	*ME*	1.00e-300	2.38e+00	1.23e-01	1.00e-300	4.25e-162	2.47e-03	9.99e-02	1.00e-300
	*STD*	1.54e-03	1.67e+00	4.27e-01	6.53e-16	3.87e+01	1.19e-01	4.66e-02	1.00e-300

**Table 11:** Comparison of average, minimum and standard deviation of error for considered PSO variants with condition 1, *I*_max_ = 500 and *D* = 50.

IW	PEC	*f*_1_	*f*_2_	*f*_3_	*f*_4_	*f*_5_
**CIW**	*AE*	2.621e-01	1.913e+00	2.626e+02	8.718e+01	3.338e+00
	*ME*	1.560e-01	1.571e+00	1.608e+02	7.324e+01	2.804e+00
	*STD*	8.869e-02	3.421e-01	5.505e+01	1.401e+01	3.286e-01
**RIW**	*AE*	7.024e-01	3.560e+00	4.866e+02	1.396e+02	4.578e+00
	*ME*	4.139e-01	2.515e+00	3.102e+02	8.621e+01	3.772e+00
	*STD*	1.592e-01	8.754e-01	1.104e+02	2.574e+01	4.289e-01
**LDIW**	*AE*	3.023e-03	7.209e-01	1.179e+02	7.728e+01	4.728e-01
	*ME*	1.560e-03	4.372e-01	4.968e+01	4.920e+01	1.489e-01
	*STD*	1.162e-03	1.636e-01	6.994e+01	1.962e+01	4.105e-01
**CHIW**	*AE*	1.144e-05	1.079e-02	8.430e+01	7.124e+01	1.943e-02
	*ME*	3.153e-06	7.664e-04	3.544e+01	5.473e+01	4.589e-03
	*STD*	6.699e-06	9.759e-03	3.968e+01	1.602e+01	2.315e-02
**FEIW-1**	*AE*	4.682e-11	9.349e-03	4.284e+01	3.814e+01	1.327e-01
	*ME*	2.378e-12	6.262e-10	1.471e-02	2.288e+01	5.449e-05
	*STD*	6.502e-11	1.398e-02	4.870e+01	1.258e+01	1.685e-01
**FEIW-2**	*AE*	9.425e-09	4.275e-03	6.528e+01	6.209e+01	8.396e-04
	*ME*	4.136e-10	4.418e-07	3.953e+01	3.980e+01	7.553e-05
	*STD*	9.711e-09	5.625e-03	3.325e+01	1.186e+01	7.821e-04
**FEIW-3**	*AE*	1.293e-04	3.436e-02	8.538e+01	6.092e+01	4.030e-02
	*ME*	1.730e-05	9.992e-03	4.550e+01	3.883e+01	1.761e-02
	*STD*	2.052e-04	1.715e-02	4.191e+01	1.510e+01	3.295e-02
**FEIW-4**	*AE*	2.348e-05	2.511e-02	6.655e+01	6.952e+01	2.603e-02
	*ME*	8.693e-06	3.454e-03	3.531e+01	4.378e+01	1.203e-02
	*STD*	1.732e-05	2.690e-02	4.544e+01	1.698e+01	1.426e-02
**FEIW-5**	*AE*	6.656e-05	2.849e-02	1.264e+02	4.329e+01	2.338e-01
	*ME*	7.866e-06	2.569e-03	3.312e+01	2.413e+01	2.359e-02
	*STD*	4.157e-05	2.351e-02	5.693e+01	1.192e+01	3.686e-01
**FEIW-6**	*AE*	5.350e-12	1.408e-02	1.130e+02	5.015e+01	8.654e-02
	*ME*	3.244e-13	1.066e-11	3.991e+01	3.383e+01	1.187e-06
	*STD*	4.591e-12	1.950e-02	6.325e+01	1.254e+01	3.320e-01

**Table 12:** Comparison of average, minimum and standard deviation of error for considered PSO variants with condition 1, *I*_max_ = 500 and *D* = 50.

IW	PEC	*f*_6_	*f*_7_	*f*_8_	*f*_9_	*f*_10_
**CIW**	*AE*	8.561e+02	1.748e+00	2.310e+01	1.813e+01	6.988e+01
	*ME*	4.180e+02	4.218e-01	1.629e+01	1.217e+01	4.365e+01
	*STD*	2.330e+02	9.425e-01	5.145e+00	4.721e+00	2.392e+01
**RIW**	*AE*	2.959e+03	1.930e+00	7.185e+01	2.061e+01	1.843e+02
	*ME*	2.017e+03	8.655e-01	3.722e+01	1.636e+01	8.530e+01
	*STD*	8.759e+02	7.457e-01	1.643e+01	4.073e+00	6.178e+01
**LDIW**	*AE*	8.385e+00	4.898e-01	2.041e-01	9.920e+00	7.967e+00
	*ME*	4.887e+00	9.144e-03	8.974e-02	6.328e+00	1.589e+00
	*STD*	2.928e+00	5.099e-01	1.188e-01	2.772e+00	4.123e+00
**CHIW**	*AE*	3.702e-02	6.486e-01	1.050e-03	8.462e+00	2.209e+00
	*ME*	2.664e-03	1.683e-05	2.423e-04	6.736e+00	7.143e-01
	*STD*	3.409e-02	1.114e+00	7.803e-04	1.162e+00	2.176e+00
**FEIW-1**	*AE*	1.194e-07	6.058e-02	3.043e-09	1.140e+01	1.687e+00
	*ME*	4.841e-09	6.031e-11	1.503e-10	6.358e+00	6.667e-01
	*STD*	2.135e-07	1.599e-01	4.426e-09	3.195e+00	1.834e+00
**FEIW-2**	*AE*	4.578e-05	9.453e-01	4.280e-07	8.846e+00	1.713e+00
	*ME*	4.183e-06	7.767e-09	2.390e-08	5.997e+00	6.667e-01
	*STD*	7.370e-05	1.151e+00	3.556e-07	1.755e+00	1.479e+00
**FEIW-3**	*AE*	3.266e-01	6.103e-01	7.833e-03	1.046e+01	4.309e+00
	*ME*	2.578e-02	1.182e-04	1.812e-03	7.441e+00	8.101e-01
	*STD*	1.772e-01	8.360e-01	6.853e-03	2.503e+00	2.911e+00
**FEIW-4**	*AE*	9.797e-02	9.640e-01	1.802e-03	8.627e+00	2.545e+00
	*ME*	7.339e-03	3.836e-05	3.546e-04	5.898e+00	6.958e-01
	*STD*	6.808e-02	1.278e+00	1.030e-03	2.208e+00	2.747e+00
**FEIW-5**	*AE*	2.533e-01	4.409e-02	5.876e-03	1.775e+01	4.155e+00
	*ME*	6.610e-02	3.795e-05	1.456e-03	1.098e+01	7.191e-01
	*STD*	2.474e-01	1.681e-01	4.898e-03	4.606e+00	2.902e+00
**FEIW-6**	*AE*	1.570e-08	5.695e-01	5.356e-10	7.939e+00	1.991e+00
	*ME*	1.751e-10	1.441e-13	1.054e-11	5.717e+00	6.667e-01
	*STD*	2.443e-08	6.986e-01	6.355e-10	1.814e+00	2.007e+00

**Table 13:** Comparison of average, minimum and standard deviation of error for considered PSO variants with condition 1, *I*_max_ = 1000 and *D* = 50.

IW	PEC	*f*_1_	*f*_2_	*f*_3_	*f*_4_	*f*_5_
**CIW**	*AE*	7.057e-02	1.255e+00	2.095e+02	7.707e+01	2.096e+00
	*ME*	3.264e-02	1.080e+00	8.116e+01	5.077e+01	1.482e+00
	*STD*	1.808e-02	1.279e-01	6.737e+01	1.655e+01	4.674e-01
**RIW**	*AE*	4.322e-01	2.286e+00	2.901e+02	1.167e+02	3.789e+00
	*ME*	2.248e-01	1.474e+00	1.625e+02	6.211e+01	3.327e+00
	*STD*	1.401e-01	5.163e-01	7.685e+01	2.848e+01	3.676e-01
**LDIW**	*AE*	5.530e-06	8.523e-03	6.976e+01	6.946e+01	1.052e-02
	*ME*	1.810e-06	1.249e-03	4.066e+01	4.082e+01	4.378e-03
	*STD*	3.780e-06	5.180e-03	2.905e+01	1.307e+01	4.197e-03
**CHIW**	*AE*	8.810e-11	4.926e-03	7.032e+01	7.177e+01	4.230e-05
	*ME*	5.580e-12	1.380e-08	4.200e+01	4.577e+01	1.380e-05
	*STD*	8.770e-11	7.324e-03	4.013e+01	1.920e+01	2.640e-05
**FEIW-1**	*AE*	3.380e-20	1.841e-02	1.067e+01	4.298e+01	3.670e-05
	*ME*	2.340e-22	1.00e-310	5.397e-03	2.487e+01	2.380e-09
	*STD*	4.800e-20	3.966e-02	2.343e+01	1.301e+01	9.830e-05
**FEIW-2**	*AE*	9.450e-17	8.704e-03	7.484e+01	7.648e+01	3.540e-08
	*ME*	3.290e-19	1.550e-15	4.214e+01	4.179e+01	3.690e-09
	*STD*	1.450e-16	8.420e-03	2.920e+01	1.649e+01	3.980e-08
**FEIW-3**	*AE*	5.300e-09	6.564e-03	8.309e+01	6.493e+01	3.880e-04
	*ME*	9.080e-10	1.880e-07	4.545e+01	3.582e+01	1.310e-04
	*STD*	7.330e-09	8.641e-03	4.054e+01	1.666e+01	3.750e-04
**FEIW-4**	*AE*	3.960e-10	6.561e-03	5.650e+01	6.865e+01	1.430e-04
	*ME*	3.890e-11	3.020e-08	4.470e+01	5.373e+01	3.400e-05
	*STD*	4.440e-10	1.038e-02	2.187e+01	9.646e+00	3.040e-04
**FEIW-5**	*AE*	1.820e-09	6.243e-03	8.025e+01	4.877e+01	1.353e-03
	*ME*	3.940e-10	7.390e-08	3.594e+01	3.085e+01	5.960e-05
	*STD*	1.760e-09	7.140e-03	3.691e+01	1.256e+01	2.769e-03
**FEIW-6**	*AE*	8.500e-22	5.419e-03	1.066e+02	5.008e+01	9.130e-10
	*ME*	2.120e-25	1.00e-310	4.396e+01	2.985e+01	1.030e-11
	*STD*	1.590e-21	7.512e-03	4.096e+01	1.589e+01	2.780e-09

**Table 14:** Comparison of average, minimum and standard deviation of error for considered PSO variants with condition 1, *I*_max_ = 1000 and *D* = 50.

IW	PEC	*f*_6_	*f*_7_	*f*_8_	*f*_9_	*f*_10_
**CIW**	*AE*	2.337e+02	5.300e-01	5.380e+00	8.877e+00	2.449e+01
	*ME*	1.252e+02	1.002e-01	2.690e+00	6.636e+00	9.647e+00
	*STD*	1.088e+02	8.105e-01	2.161e+00	1.121e+00	8.589e+00
**RIW**	*AE*	1.508e+03	1.565e+00	3.334e+01	1.155e+01	9.211e+01
	*ME*	9.102e+02	6.392e-01	1.809e+01	8.175e+00	5.342e+01
	*STD*	4.950e+02	8.229e-01	7.235e+00	1.754e+00	3.208e+01
**LDIW**	*AE*	2.356e-02	3.037e-02	6.840e-04	3.377e+00	2.313e+00
	*ME*	3.943e-03	1.220e-05	6.870e-05	2.171e+00	6.826e-01
	*STD*	1.698e-02	1.173e-01	5.610e-04	7.384e-01	2.590e+00
**CHIW**	*AE*	2.370e-07	6.544e-01	5.960e-09	2.651e+00	2.265e+00
	*ME*	3.590e-08	7.160e-11	2.720e-10	2.024e+00	6.667e-01
	*STD*	1.710e-07	7.466e-01	6.120e-09	6.250e-01	2.380e+00
**FEIW-1**	*AE*	3.380e-16	2.726e-01	2.410e-18	2.661e+00	1.046e+00
	*ME*	6.330e-19	1.520e-21	1.380e-20	1.664e+00	6.667e-01
	*STD*	9.840e-16	4.136e-01	4.040e-18	4.824e-01	1.011e+00
**FEIW-2**	*AE*	8.620e-13	7.939e-01	4.040e-15	3.377e+00	1.382e+00
	*ME*	1.360e-15	3.750e-18	1.140e-17	2.273e+00	6.667e-01
	*STD*	2.050e-12	8.258e-01	7.460e-15	8.496e-01	1.976e+00
**FEIW-3**	*AE*	1.860e-05	5.030e-01	2.840e-07	3.099e+00	2.131e+00
	*ME*	2.230e-06	1.030e-09	2.160e-08	1.411e+00	6.667e-01
	*STD*	2.790e-05	8.337e-01	3.400e-07	6.550e-01	2.883e+00
**FEIW-4**	*AE*	1.680e-06	3.272e-01	1.750e-08	2.366e+00	2.232e+00
	*ME*	1.040e-07	8.510e-11	3.340e-09	1.406e+00	6.667e-01
	*STD*	1.930e-06	5.106e-01	1.330e-08	4.775e-01	2.164e+00
**FEIW-5**	*AE*	5.880e-06	1.260e-01	9.030e-08	4.973e+00	1.528e+00
	*ME*	4.500e-07	1.470e-09	1.760e-08	3.454e+00	6.667e-01
	*STD*	5.310e-06	2.066e-01	1.000e-07	9.977e-01	2.146e+00
**FEIW-6**	*AE*	2.160e-19	2.423e-01	1.520e-20	3.151e+00	1.173e+00
	*ME*	2.480e-21	1.740e-23	6.290e-23	2.241e+00	6.667e-01
	*STD*	3.230e-19	3.377e-01	2.380e-20	7.677e-01	8.747e-01

### 6.2 Comparison Analysis of IW Strategies

According to the numerical results obtained from this study (Tables 5–14), we can compare IW strategies with each other based on any benchmark function. For each problem and each PEC, the best and worst IW strategies have been determined in Tables 15–22. The following notation is used in these tables:

**Table 15:** Best and worst IW strategies for each benchmark function in terms of success rate, average and minimum number of iterations of successful runs according to Table 5.

PEC	Case	*f*_1_	*f*_2_	*f*_3_	*f*_4_	*f*_5_	*f*_6_	*f*_7_	*f*_8_	*f*_9_	*f*_10_
***SR***	Best	S-FEIW	FEIW-3	FEIW-1	S-FEIW	S-FEIW	S-FEIW	S-FEIW	S-FEIW	S-FEIW	FEIW-6
	Worst	RIW	RIW	LDIW	S-FEIW	S-IW	RIW	RIW	RIW	S-IW	FEIW-4
***ANS***	Best	FEIW-1	FEIW-5	FEIW-5	S-FEIW	FEIW-1	FEIW-1	FEIW-1	FEIW-1	FEIW-6	FEIW-6
	Worst	RIW	RIW	LDIW	S-FEIW	S-IW	RIW	RIW	RIW	S-IW	LDIW
***MNS***	Best	FEIW-1	FEIW-1	FEIW-1	S-FEIW	FEIW-1	FEIW-1	FEIW-1	FEIW-1	FEIW-6	FEIW-1
	Worst	RIW	RIW	LDIW	S-FEIW	S-IW	RIW	RIW	RIW	S-IW	LDIW

**Table 16:** Best and worst IW strategies for each benchmark function in terms of success rate, average and minimum number of iterations of successful runs according to Table 6.

PEC	Case	*f*_11_	*f*_12_	*f*_13_	*f*_14_	*f*_15_	*f*_16_	*f*_17_	*f*_18_
***SR***	Best	S-FEIW	S-FEIW	FEIW-1	S-FEIW	FEIW-3	S-FEIW	S-FEIW	S-FEIW
	Worst	S-IW	S-IW	GLBIW	S-IW	FEIW-2	S-IW	S-IW	S-IW
***ANS***	Best	FEIW-1	FEIW-1	FEIW-5	FEIW-1	FEIW-1	FEIW-5	FEIW-1	FEIW-1
	Worst	S-IW	S-IW	GLBIW	S-IW	FEIW-5	S-IW	S-IW	S-IW
***MNS***	Best	FEIW-1	FEIW-1	FEIW-1	FEIW-1	FEIW-1	FEIW-6	FEIW-1	FEIW-1
	Worst	S-IW	S-IW	GLBIW	S-IW	AIW	S-IW	S-IW	S-IW

**Table 17:** Best and worst IW strategies for each benchmark function in terms of success rate, average and minimum number of iterations of successful runs according to Table 6.

PEC	Case	*f*_19_	*f*_20_	*f*_21_	*f*_22_	*f*_23_	*f*_24_	*f*_25_	*f*_26_
***SR***	Best	S-FEIW	FEIW-4	FEIW-1	S-FEIW	FEIW-2	FEIW-4	FEIW-3	S-FEIW
	Worst	GLBIW	GLBIW	S-FEIW	S-IW	S-IW	AIW	AIW	GLBIW
***ANS***	Best	FEIW-1	FEIW-5	FEIW-1	FEIW-1	FEIW-1	FEIW-5	FEIW-5	FEIW-1
	Worst	AIW	FEIW-1	S-FEIW	S-IW	S-IW	AIW	AIW	AIW
***MNS***	Best	FEIW-1	GLBIW	FEIW-1	FEIW-1	FEIW-1	FEIW-1	FEIW-6	FEIW-1
	Worst	FEIW-4	FEIW-2	S-FEIW	S-IW	S-IW	FEIW-4	AIW	FEIW-4

**Table 18:** Best and worst IW strategies for each benchmark function in terms of average, minimum and standard deviation of error according to Tables 7 and 8.

PEC	Case	*F*_1_	*f*_2_	*f*_3_	*f*_4_	*f*_5_	*f*_6_	*f*_7_	*f*_8_	*f*_9_	*f*_10_
***AE***	Best	FEIW-1	FEIW-2	FEIW-1	RIW	S-FEIW	FEIW-1	S-FEIW	FEIW-1	FEIW-6	FEIW-6
	Worst	LDIW	LDIW	CHIW	FEIW-5	LDIW	LDIW	RIW	LDIW	LDIW	LDIW
***ME***	Best	FEIW-1	FEIW-3	FEIW-6	S-FEIW	S-FEIW	FEIW-1	FEIW-1	FEIW-1	FEIW-6	FEIW-6
	Worst	CHIW	RIW	CHIW	FEIW-5	CHIW	LDIW	LDIW	LDIW	LDIW	S-IW
***STD***	Best	FEIW-1	FEIW-5	CHIW	FEIW-5	S-FEIW	FEIW-1	S-FEIW	FEIW-1	FEIW-6	CHIW
	Worst	LDIW	LDIW	FEIW-6	LDIW	LDIW	LDIW	RIW	LDIW	LDIW	S-FEIW

**Table 19:** Best and worst IW strategies for each benchmark function in terms of average, minimum and standard deviation of error according to Table 9.

PEC	Case	*f*_11_	*f*_12_	*f*_13_	*f*_14_	*f*_15_	*f*_16_	*f*_17_	*f*_18_
***AE***	Best	FEIW-6	FEIW-1	FEIW-1	FEIW-6	FEIW-3	S-FEIW	S-FEIW	S-FEIW
	Worst	GLBIW	GLBIW	GLBIW	GLBIW	FEIW-6	GLBIW	GLBIW	GLBIW
***ME***	Best	FEIW-6	FEIW-6	S-FEIW	FEIW-6	FEIW-3	S-FEIW	S-FEIW	S-FEIW
	Worst	GLBIW	GLBIW	GLBIW	GLBIW	S-FEIW	GLBIW	GLBIW	GLBIW
***STD***	Best	FEIW-6	FEIW-6	FEIW-1	FEIW-6	AIW	S-IW	S-FEIW	S-FEIW
	Worst	GLBIW	GLBIW	GLBIW	GLBIW	FEIW-6	GLBIW	GLBIW	GLBIW

**Table 20:** Best and worst IW strategies for each benchmark function in terms of average, minimum and standard deviation of error according to Table 10.

PEC	Case	*f*_19_	*f*_20_	*f*_21_	*f*_22_	*f*_23_	*f*_24_	*f*_25_	*f*_26_
***AE***	Best	S-FEIW	FEIW-1	FEIW-1	S-FEIW	FEIW-4	FEIW-1	FEIW-1	S-FEIW
	Worst	NEIW	FEIW-5	GLBIW	GLBIW	FEIW-6	AIW	AIW	GLBIW
***ME***	Best	S-FEIW	FEIW-1	FEIW-4	S-FEIW	FEIW-6	FEIW-2	S-FEIW	S-FEIW
	Worst	AIW	GLBIW	EDIW	GLBIW	GLBIW	GLBIW	S-FEIW	AIW
***STD***	Best	S-FEIW	FEIW-4	FEIW-1	S-FEIW	FEIW-4	EDIW	FEIW-4	S-FEIW
	Worst	NEIW	FEIW-1	GLBIW	GLBIW	FEIW-1	FEIW-6	FEIW-5	GLBIW

**Table 21:** Best and worst IW strategies for each benchmark function in terms of average, minimum and standard deviation of error according to Tables 11 and 12.

PEC	Case	*f*_1_	*f*_2_	*f*_3_	*f*_4_	*f*_5_	*f*_6_	*f*_7_	*f*_8_	*f*_9_	*f*_10_
***AE***	Best	FEIW-6	FEIW-2	FEIW-1	FEIW-1	FEIW-2	FEIW-6	FEIW-5	FEIW-6	FEIW-6	FEIW-1
	Worst	RIW	RIW	RIW	RIW	RIW	RIW	RIW	RIW	RIW	RIW
***ME***	Best	FEIW-6	FEIW-6	FEIW-1	FEIW-1	FEIW-6	FEIW-6	FEIW-6	FEIW-6	FEIW-6	S-FEIW
	Worst	RIW	RIW	RIW	RIW	RIW	RIW	RIW	RIW	RIW	RIW
***STD***	Best	FEIW-6	FEIW-2	FEIW-2	S-FEIW	FEIW-2	FEIW-6	FEIW-1	FEIW-6	CHIW	FEIW-2
	Worst	RIW	RIW	RIW	RIW	RIW	RIW	FEIW-4	RIW	CIW	RIW

**Table 22:** Best and worst IW strategies for each benchmark function in terms of average, minimum and standard deviation of error according to Tables 13 and 14.

PEC	Case	*f*_1_	*f*_2_	*f*_3_	*f*_4_	*f*_5_	*f*_6_	*f*_7_	*f*_8_	*f*_9_	*f*_10_
***AE***	Best	FEIW-6	CHIW	FEIW-1	FEIW-1	FEIW-6	FEIW-6	LDIW	FEIW-6	FEIW-4	FEIW-1
	Worst	RIW	RIW	RIW	RIW	RIW	RIW	RIW	RIW	RIW	RIW
***ME***	Best	FEIW-6	S-FEIW	FEIW-1	FEIW-1	FEIW-6	FEIW-6	FEIW-6	FEIW-6	S-FEIW	S-FEIW
	Worst	RIW	RIW	RIW	RIW	RIW	RIW	RIW	RIW	RIW	RIW
***STD***	Best	FEIW-6	LDIW	FEIW-4	FEIW-4	FEIW-6	FEIW-6	LDIW	FEIW-6	FEIW-4	FEIW-6
	Worst	RIW	RIW	RIW	RIW	CIW	RIW	FEIW-3	RIW	RIW	RIW

S-IW indicates several inertia weights except variations of FEIW. Also S-FEIW indicates several inertia weights including some variations of FEIW. For example in Table 17, the worst IW strategies for Pinter function (*f*_23_) in terms of *ANS*, are GLBIW and AIW, also in Table 20, the best IW strategies for Quintic function (*f*_22_) in terms of *AE*, are FEIW-3, FEIW-5 and NEIW. Thus the notations S-IW and S-FEIW are used in the *f*_23_ and *f*_22_ columns of Tables 17 and 20, respectively. It can be seen from Tables 15–22 that variations of FEIW emerge as best performers. Let $N_{\text{PEC}}^{T}$ be the number of benchmark functions in table *T* (15 ≤ *T* ≤ 22) which achieve the best result with variations of FEIW strategy in terms of PEC. Also let $N_{{}_{\text{Total}}}^{T}$ be the total number of benchmark functions in table *T*. If we define $P_{\text{PEC}}^{T}=\frac{N_{\text{PEC}}^{T}}{N_{\text{Total}}^{T}}\times 100$ then $P_{\text{PEC}}^{T}$ is the percentage of successful FEIW strategies in terms of PEC among all benchmark functions in table *T*. Using this definition, we can summarize Tables 15–22 in Table 23. For example in this table, $P_{AE}^{18}=90\%$, i.e., 90% of IW strategies that can provide the best average error performance for benchmark functions, are variations of FEIW. From Table 23, it could be concluded that FEPSO seems to be more efficient and has good convergence compared to other IW strategies. In the next subsection, we will show that statistical tests confirm that the variations of FEIW significantly improves results.

**Table 23:** Summary of results of Tables 15–2,2.

T	$P_{AE}^{T}$	$P_{ME}^{T}$	$P_{STD}^{T}$	$P_{SR}^{T}$	$P_{ANS}^{T}$	$P_{MNS}^{T}$
**15**	---	---	---	100%	100%	100%
**16 and 17**	---	---	---	100%	100%	94%
**18**	90%	100%	80%	---	---	---
**19 and 20**	100%	100%	81%	---	---	---
**21**	100%	100%	90%	---	---	---
**22**	80%	100%	80%	---	---	---

### 6.3 Statistical analysis of numerical results

In this section, the numerical results obtained using FEIW strategy and other strategies are statistically analyzed based on non-parametric tests as: Wilcoxon test; Friedman test and Bonferroni-Dunn test [35–37]. The Wilcoxon test performs pair wise comparison of variants while Bonferroni-Dunn test detects the significant differences among all variants. Because of nature of numerical results, the logarithmic scale of average, minimum and standard deviation of error are used for statistical tests.

#### 6.3.1 Wilcoxon sign rank test

Wilcoxon sign rank test is nonparametric statistically hypothesis test which can be used as an alternative to the paired t-test when the results cannot be assumed to be normally distributed. The results for Wilcoxon’s test are summarized as *R*^**+**^ and *R*^**−**^, which represent the sum of positive and negative ranks of an algorithm in comparison to other algorithms in the column. During statistical analysis on Table 5, we have considered two performance criteria, average and minimum number of iterations of successful runs, which evaluate the convergence speed of a given algorithm. Table 24 comprises results of wilcoxon signed rank test for these two performance criteria taken *I*_max_ = 1000 and *D* = 10. Table 24 shows that the variations of FEIW win over other strategies in 23 of 24 tests in terms of average number of iterations of successful runs. Also the p-value in most of the cases is less than 0.01. Thus in terms of average number of iterations of successful runs, all the six variations of FEIW are significantly better than CIW, RIW, LDIW and CHIW. According to Table 24, this is true for minimum number of iterations of successful runs. Therefore the wilcoxon sign rank test on Table 5 clearly proves the superiority of FEIW over other IW models in terms of convergence speed. Table 25 shows the results for wilcoxon signed rank test for average and minimum number of iterations of successful runs according to Table 6. Table 25 shows that FEIW-1, FEIW-5 and FEIW-6 win over GLBIW, AIW, NEIW and EDIW in the all cases and also the p-value is less than 0.01 and thus these three variations of FEIW are significantly better than other IW strategies in terms of convergence speed. With applying statistical analysis on Tables 7 and 8, we can evaluate the solution precision of FEPSO algorithm. Table 26 comprises results of wilcoxon signed rank test for average and minimum error taken for *I*_max_ = 1000 and *D* = 10. Table 26 shows that except in FEIW-4, the other variations of FEIW win over other strategies in most of the cases with p-value<0.05. Thus in terms of average and minimum error, FEIW is significantly better than CIW, RIW, LDIW and CHIW. Therefore the wilcoxon sign rank test on Tables 7 and 8 clearly proves the superiority of FEIW over other IW models in terms of solution precision. Table 27 shows the results for wilcoxon signed rank test for average and minimum error according to Tables 9 and 10. The observation of results in Table 27 confirms that FEIW-1 wins in the all cases with p-value less than 0.05 and is significantly better than GLBIW, AIW, NEIW and EDIW. Using wilcoxon signed rank test from Tables 11 and 12, the solution precision of FEPSO algorithm for *I*_max_ = 500 and *D* = 50 can be evaluated. Table 28 contains results of this test for average and minimum error. In terms of average error, all the variations of FEIW win over CIW, RIW and LDIW strategies in all the cases with p-value<0.05. Also FEIW-2 wins over CHIW strategy in all the cases with p-value< 0.05. In terms of minimum error, all the variations of FEIW win over CIW, RIW and LDIW strategies in all the cases with p-value<0.05. Also FEIW-1, FEIW-2 and FEIW-6 win over CHIW strategy in all the cases with p-value<0.05. Thus in terms of average and minimum error, FEIW is significantly better than CIW, RIW, LDIW and CHIW. Therefore the wilcoxon sign rank test on Tables 11 and 12 confirms the superiority of FEIW over other IW strategies in terms of solution precision. With applying wilcoxon signed rank test from Tables 13 and 14, the solution precision of FEPSO algorithm for, *I*_max_ = 1000 and *D* = 50 can be evaluated. Table 29 contains results of this test for average and minimum error. In terms of average error, all the variations of FEIW win over CIW and RIW strategies in all the cases with p-value<0.05. Also FEIW-4 wins over LDIW strategy and FEIW-1 and FEIW-6 win over CHIW strategy in all the cases with p-value<0.05. In terms of minimum error, all the variations of FEIW win over CIW, RIW and LDIW strategies in all the cases with p-value< 0.05. Also FEIW-1, FEIW-2 and FEIW-6 win over CHIW strategy in all the cases with p-value< 0.05. Thus in terms of average and minimum error, FEIW is significantly better than CIW, RIW, LDIW and CHIW. Therefore the wilcoxon sign rank test on Tables 13 and 14 confirms the superiority of FEIW over other IW strategies in terms of solution precision.

**Table 24:** Wilcoxon-ranks and p-value on the average and minimum number of iterations of successful runs according to Table 5.

Mode		Average Iterations				Minimum Iterations			
Algorithm	Statistical measures	CIW	RIW	LDIW	CHIW	CIW	RIW	LDIW	CHIW
**FEIW-1**	*R*^+^	45	45	45	45	45	45	45	45
	*R*^−^	0	0	0	0	0	0	0	0
	p-value	0.008	0.008	0.008	0.008	0.008	0.008	0.008	0.008
**FEIW-2**	*R*^+^	45	45	45	45	44	45	45	43
	*R*^−^	0	0	0	0	1	0	0	2
	p-value	0.008	0.008	0.008	0.008	0.011	0.008	0.008	0.015
**FEIW-3**	*R*^+^	45	45	45	45	45	45	45	45
	*R*^−^	0	0	0	0	0	0	0	0
	p-value	0.008	0.008	0.008	0.008	0.008	0.008	0.008	0.008
**FEIW-4**	*R*^+^	44	42	45	4	34	43	45	45
	*R*^−^	1	3	0	41	11	2	0	0
	p-value	0.011	0.021	0.008	0.028	0.173	0.015	0.008	0.008
**FEIW-5**	*R*^+^	45	45	45	45	45	45	45	45
	*R*^−^	0	0	0	0	0	0	0	0
	p-value	0.008	0.008	0.008	0.008	0.008	0.008	0.008	0.008
**FEIW-6**	*R*^+^	45	45	45	45	45	45	45	45
	*R*^−^	0	0	0	0	0	0	0	0
	p-value	0.008	0.008	0.008	0.008	0.008	0.008	0.008	0.008

**Table 25:** Wilcoxon-ranks and p-value on the average and minimum number of iterations of successful runs according to Table 6.

Mode		Average Iterations				Minimum Iterations			
Algorithm	Statistical measures	GLBIW	AIW	NEIW	EDIW	GLBIW	AIW	NEIW	EDIW
**FEIW-1**	*R*^+^	132	135	134	126	135	135	136	136
	*R*^−^	4	1	2	10	1	1	0	0
	p-value	0.001	0.001	0.001	0.003	0.001	0.001	0.000	0.000
**FEIW-2**	*R*^+^	112	119	118	109	108	117	119	27
	*R*^−^	8	1	2	11	12	3	1	93
	p-value	0.003	0.001	0.001	0.005	0.006	0.001	0.001	0.061
**FEIW-3**	*R*^+^	109	118	104	21	113	119	120	71.5
	*R*^−^	11	2	16	57	7	1	0	48.5
	p-value	0.005	0.001	0.012	0.157	0.003	0.001	0.001	0.514
**FEIW-4**	*R*^+^	101	117	0	0	99	109	2	0
	*R*^−^	19	3	120	120	21	11	118	120
	p-value	0.020	0.001	0.001	0.001	0.027	0.005	0.001	0.001
**FEIW-5**	*R*^+^	131	135	121	121	135	135	136	136
	*R*^−^	5	1	15	15	1	1	0	0
	p-value	0.001	0.001	0.006	0.006	0.001	0.001	0.000	0.000
**FEIW-6**	*R*^+^	135	136	134	128	135	135	136	136
	*R*^−^	1	0	2	8	1	1	0	0
	p-value	0.001	0.001	0.001	0.002	0.001	0.001	0.000	0.000

**Table 26:** Wilcoxon-ranks and p-value on the average and minimum error according to Tables 7 and 8.

Mode		Average Error				Minimum Error			
Algorithm	Statistical measures	CIW	RIW	LDIW	CHIW	CIW	RIW	LDIW	CHIW
**FEIW-1**	*R*^+^	46	54	49	48	54	55	55	55
	*R*^−^	9	1	6	7	1	0	0	0
	p-value	0.059	0.007	0.028	0.037	0.007	0.005	0.005	0.005
**FEIW-2**	*R*^+^	51	53	55	55	52	53	55	55
	*R*^−^	4	2	0	0	3	2	0	0
	p-value	0.017	0.009	0.005	0.005	0.013	0.009	0.005	0.005
**FEIW-3**	*R*^+^	51	53	53	53	54	54	54	55
	*R*^−^	4	2	2	2	1	1	1	0
	p-value	0.017	0.009	0.009	0.009	0.007	0.007	0.007	0.005
**FEIW-4**	*R*^+^	2	48	55	17	0	11	50	21
	*R*^−^	53	7	0	38	55	34	5	34
	p-value	0.009	0.037	0.005	0.285	0.005	0.173	0.022	0.508
**FEIW-5**	*R*^+^	52	54	54	54	51	53	52	53
	*R*^−^	3	1	1	1	4	2	3	2
	p-value	0.013	0.007	0.007	0.007	0.017	0.009	0.013	0.009
**FEIW-6**	*R*^+^	52	54	54	54	52	54	53	54
	*R*^−^	3	1	1	1	3	1	2	1
	p-value	0.013	0.007	0.007	0.007	0.013	0.007	0.009	0.007

**Table 27:** Wilcoxon-ranks and p-value on the average and minimum error according to Tables 9 and 10.

Mode		Average Error				Minimum Error			
Algorithm	Statistical measures	GLBIW	AIW	NEIW	EDIW	GLBIW	AIW	NEIW	EDIW
**FEIW-1**	*R*^+^	136	134	67	66	105	120	36	36
	*R*^−^	0	2	11	0	0	0	0	0
	p-value	0.000	0.001	0.028	0.003	0.001	0.001	0.012	0.012
**FEIW-2**	*R*^+^	135	133	42	34	118	116	27	35
	*R*^−^	1	3	49	44	2	4	1	1
	p-value	0.001	0.001	0.807	0.695	0.001	0.001	0.028	0.017
**FEIW-3**	*R*^+^	135	130	22	21	119	118	8	10
	*R*^−^	1	6	56	70	1	2	28	26
	p-value	0.001	0.001	0.182	0.087	0.001	0.001	0.161	0.263
**FEIW-4**	*R*^+^	136	134	38	30	119	118	10	6
	*R*^−^	0	2	53	48	1	2	26	22
	p-value	0.000	0.001	0.600	0.480	0.001	0.001	0.263	0.176
**FEIW-5**	*R*^+^	131	124	14	19	120	117	5	7
	*R*^−^	5	12	77	86	0	3	31	29
	p-value	0.001	0.004	0.028	0.035	0.001	0.001	0.069	0.123
**FEIW-6**	*R*^+^	126	121.5	42	36	115	114	24	29
	*R*^−^	10	14.5	63	69	5	6	12	16
	p-value	0.003	0.006	0.510	0.300	0.002	0.002	0.401	0.441

**Table 28:** Wilcoxon-ranks and p-value on the average and minimum error according to Tables 11 and 12.

Mode		Average Error				Minimum Error			
Algorithm	Statistical measures	CIW	RIW	LDIW	CHIW	CIW	RIW	LDIW	CHIW
**FEIW-1**	*R*^+^	55	55	54	46	55	55	54	55
	*R*^−^	0	0	1	9	0	0	1	0
	p-value	0.005	0.005	0.007	0.059	0.005	0.005	0.007	0.005
**FEIW-2**	*R*^+^	55	55	51	49	55	55	55	53
	*R*^−^	0	0	4	6	0	0	0	2
	p-value	0.005	0.005	0.017	0.028	0.005	0.005	0.005	0.009
**FEIW-3**	*R*^+^	55	55	52	5	55	55	53	4
	*R*^−^	0	0	3	50	0	0	2	51
	p-value	0.005	0.005	0.013	0.022	0.005	0.005	0.009	0.017
**FEIW-4**	*R*^+^	55	55	51	6	55	55	55	10
	*R*^−^	0	0	4	49	0	0	0	45
	p-value	0.005	0.005	0.017	0.028	0.005	0.005	0.005	0.074
**FEIW-5**	*R*^+^	55	55	51	12	55	55	53	7
	*R*^−^	0	0	4	43	0	0	2	48
	p-value	0.005	0.005	0.017	0.114	0.005	0.005	0.009	0.037
**FEIW-6**	*R*^+^	55	55	53	39	55	55	55	53
	*R*^−^	0	0	2	16	0	0	0	2
	p-value	0.005	0.005	0.009	0.241	0.005	0.005	0.005	0.009

**Table 29:** Wilcoxon-ranks and p-value on the average and minimum error according to Tables 13 and 14.

Mode		Average Error				Minimum Error			
Algorithm	Statistical measures	CIW	RIW	LDIW	CHIW	CIW	RIW	LDIW	CHIW
**FEIW-1**	*R*^+^	55	55	46	48	55	55	55	45
	*R*^−^	0	0	9	7	0	0	0	0
	p-value	0.005	0.005	0.059	0.037	0.005	0.005	0.005	0.008
**FEIW-2**	*R*^+^	53	55	34	39	55	55	47	41
	*R*^−^	2	0	11	16	0	0	8	4
	p-value	0.009	0.005	0.173	0.241	0.005	0.005	0.047	0.028
**FEIW-3**	*R*^+^	55	55	45	8	55	55	53	5
	*R*^−^	0	0	10	47	0	0	2	40
	p-value	0.005	0.005	0.074	0.047	0.005	0.005	0.009	0.038
**FEIW-4**	*R*^+^	55	55	49	16	54	55	50	4
	*R*^−^	0	0	6	39	1	0	5	41
	p-value	0.005	0.005	0.028	0.241	0.007	0.005	0.022	0.028
**FEIW-5**	*R*^+^	55	55	44	13	55	55	51	3
	*R*^−^	0	0	11	42	0	0	4	42
	p-value	0.005	0.005	0.093	0.139	0.005	0.005	0.017	0.021
**FEIW-6**	*R*^+^	55	55	46	48	55	55	50	42
	*R*^−^	0	0	9	7	0	0	5	3
	p-value	0.005	0.005	0.059	0.037	0.005	0.005	0.022	0.021

#### 6.3.2 Friedman test

The Friedman test is a non-parametric statistical test developed by the Friedman [38, 39]. The goal of this test is to determine whether there are significant differences among the algorithms considered over given sets of data. The Friedman test determines the ranks of the algorithms for each individual data set, i.e., in the minimization problems, the best performing algorithm getting minimum rank. Outcomes of Friedman test on Tables 5–14 are shown in Tables 30–35. The results of Friedman test are used to observe whether there is overall difference among IW strategies. In all tables the p-value of Friedman test is lower than the level of significance considered *α* = 0.05 and *α* = 0.01 thus there are significant differences among the observed results. The speed in obtaining the global optimum is a salient yardstick for measuring the algorithm performance. From Table 30, FEIW-1 has the best performance among all IW strategies, in terms of average and minimum number of iterations. Also FEIW-5 has the highest rank of success rate. Similarly, Table 31 shows that FEIW-1 has the best rank among all IW strategies in terms of success rate, average and minimum number of iterations. Thus with condition 2, *I*_max_ = 1000 and *D* = 10, Friedman test proves the advantage of FEIW-1 and FEIW-5 over other IW strategies in terms of convergence speed and solution precision. From Table 32, FEIW-6 and FEIW-1 have the best performance among all IW strategies, in terms of average and minimum error, respectively. Also Table 33 shows that FEIW-1 has the best rank in terms of average and minimum error. Thus with condition 1, *I*_max_ = 1000 and *D* = 10, Friedman test proves that FEIW-6 and FEIW-1 are the best strategies for better accuracy. Under condition 1, *I*_max_ = 500 and *D* = 50, from Table 34 one can observe that FEIW-1 and FEIW-6 have the highest performance since these strategies have minimum rank, in terms of average and minimum error, respectively. With condition 1, *I*_max_ = 1000 and *D* = 50, from Table 35 one can conclude that FEIW-1 is the best IW strategy in both average and minimum error test. Therefore, FEPSO significantly outperforms CIWPSO, RIWPSO, LDIWPSO, CHIWPSO, GLBIWPSO, AIWPSO, NEIWPSO and EDIWPSO in terms of solution quality and convergence rate using the Friedman test.

**Table 30:** Friedman test based on Table 5.

		Average Iterations	Minimum Iterations	Success Rate
**Results**	*N*	10	10	10
	Chisquare	75.2	76.4	27.1
	p-value	1.4 × 10^−12^	8.2 × 10^−13^	1.4 × 10^−3^
**Mean Ranking**	CIW	8.45	7.85	3.90
	RIW	8.85	9.05	2.45
	LDIW	8.35	8.55	5.85
	CHIW	6.15	5.95	6.40
	FEIW-1	**2.15**	**1.65**	5.80
	FEIW-2	4.95	5.25	6.15
	FEIW-3	4.15	4.15	6.20
	FEIW-4	6.95	7.15	5.65
	FEIW-5	2.65	3.00	**6.45**
	FEIW-6	2.35	2.40	6.15

**Table 31:** Friedman test based on Table 6.

		Average Iterations	Minimum Iterations	Success Rate
**Results**	*N*	16	16	16
	Chisquare	101.7	113.0	73.7
	p-value	6.9 × 10^−18^	3.5 × 10^−20^	2.8 × 10^−12^
**Mean Ranking**	GLBIW	8.00	7.75	2.03
	AIW	9.13	8.63	2.31
	NEIW	6.88	7.38	6.25
	EDIW	4.59	5.13	6.34
	FEIW-1	**2.09**	**1.31**	**7.38**
	FEIW-2	5.69	6.38	5.94
	FEIW-3	5.16	5.00	6.94
	FEIW-4	8.13	8.38	7.06
	FEIW-5	2.72	2.84	5.38
	FEIW-6	2.63	2.22	5.38

**Table 32:** Friedman test based on Tables 7 and 8.

		Average Error	Minimum Error	Standard deviation
**Results**	*N*	10	10	10
	Chisquare	47.7	55.5	31.0
	p-value	2.8 × 10^−7^	9.8 × 10^−9^	3 × 10^−4^
**Mean Ranking**	CIW	5.90	5.80	6.45
	RIW	7.80	7.65	7.50
	LDIW	9.20	8.55	8.40
	CHIW	7.00	8.05	5.40
	FEIW-1	3.55	**2.25**	3.85
	FEIW-2	4.35	4.30	4.85
	FEIW-3	3.95	3.65	4.85
	FEIW-4	7.10	7.75	6.90
	FEIW-5	3.45	4.15	**2.95**
	FEIW-6	**2.70**	2.85	3.85

**Table 33:** Friedman test based on Tables 9 and 10.

		Average Error	Minimum Error	Standard deviation
**Results**	*N*	16	16	16
	Chisquare	66.6	63.1	54.0
	p-value	6.9 × 10^−11^	3.3 × 10^−10^	1.8 × 10^−8^
**Mean Ranking**	GLBIW	9.28	8.94	8.41
	AIW	8.00	8.16	7.69
	NEIW	4.72	5.09	4.66
	EDIW	4.47	5.19	4.16
	FEIW-1	**2.50**	**3.63**	**2.56**
	FEIW-2	4.66	3.97	4.53
	FEIW-3	5.13	5.22	5.63
	FEIW-4	4.06	4.75	4.50
	FEIW-5	6.31	5.22	6.81
	FEIW-6	5.88	4.84	6.06

**Table 34:** Friedman test based on Tables 11 and 12.

		Average Error	Minimum Error	Standard deviation
**Results**	*N*	10	10	10
	Chisquare	64.3	75.2	48.9
	p-value	2 × 10^−10^	1.4 × 10^−12^	1.7 × 10^−7^
**Mean Ranking**	CIW	9.00	9.00	7.90
	RIW	10.0	10.0	9.30
	LDIW	7.10	7.50	7.60
	CHIW	4.00	4.70	3.90
	FEIW-1	**2.60**	2.10	3.30
	FEIW-2	3.10	3.30	**2.50**
	FEIW-3	5.90	6.50	5.50
	FEIW-4	4.80	4.80	5.50
	FEIW-5	5.60	5.30	5.90
	FEIW-6	2.90	**1.80**	3.60

**Table 35:** Friedman test based on Tables 13 and 14.

		Average Error	Minimum Error	Standard deviation
**Results**	*N*	10	10	10
	Chisquare	55.0	66.7	50.1
	p-value	1.2 × 10^−8^	6.8 × 10^−11^	10^−7^
**Mean Ranking**	CIW	8.80	8.90	8.70
	RIW	10.0	10.0	9.70
	LDIW	6.25	6.90	5.30
	CHIW	4.50	4.30	4.80
	FEIW-1	**2.70**	**2.05**	**3.10**
	FEIW-2	4.95	4.00	4.50
	FEIW-3	5.75	5.90	7.00
	FEIW-4	4.35	5.10	4.00
	FEIW-5	5.00	5.40	4.80
	FEIW-6	2.70	2.45	**3.10**

#### 6.3.3 Bonferroni-Dunn test

Here we have employed Bonferroni-Dunn test [40] to detect significant differences for the considered variants. The Bonferroni-Dunn test is used to compare an IW strategy with all the other strategies. The performance of two strategies is significantly different if the corresponding mean ranks differ by at least the critical difference (CD):

$$CD_{\alpha }=q_{\alpha }\sqrt{\frac{N_{i}(N_{i}+1)}{6N_{f}}}$$ (41)

where *N*_*i*_ and *N*_*f*_ are number of IW strategies and benchmark functions, respectively. Also critical values *q*_*α*_ at the probability level *α* is given in [35] as follows

$$q_{0.05}=2.773\begin{array}{ccc} & , & \end{array}q_{0.1}=2.539$$ (42)

Using Eqs (41) and (42) critical difference for Bonferroni-Dunn test after the Friedman test is as follows

$$CD_{0.05}=3.7547\begin{array}{ccc} & , & \end{array}CD_{0.1}=3.4378$$ (43)

**Fig 3:**
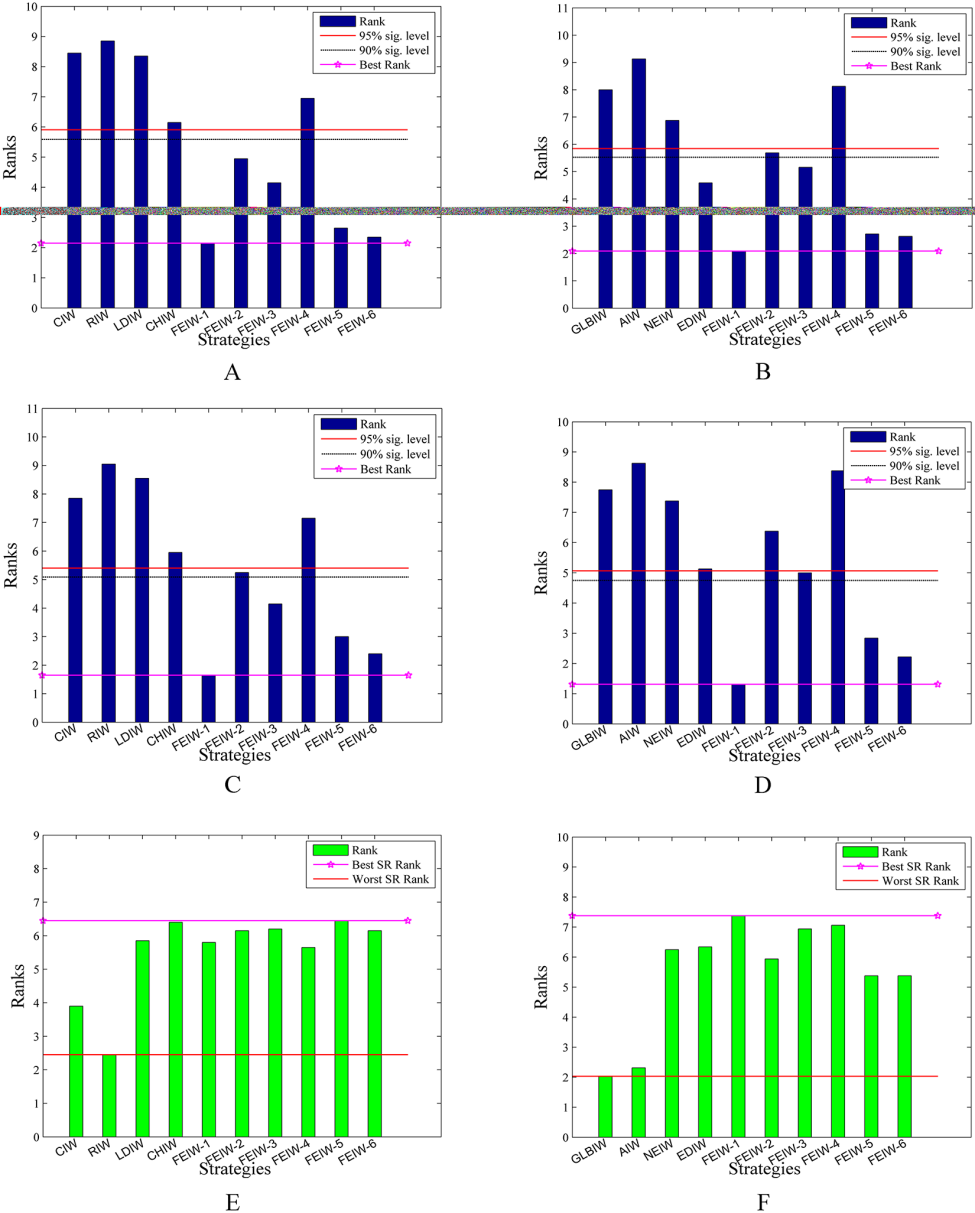
Bonferroni-Dunn bar chart. (A) Average iterations based on Table 5. (B) Average iterations based on Table 6. (C) Minimum iterations based on Table 5. (D) Minimum iterations based on Table 6. (E) Success rate based on Table 5. (F) Success rate based on Table 6.

**Fig 4:**
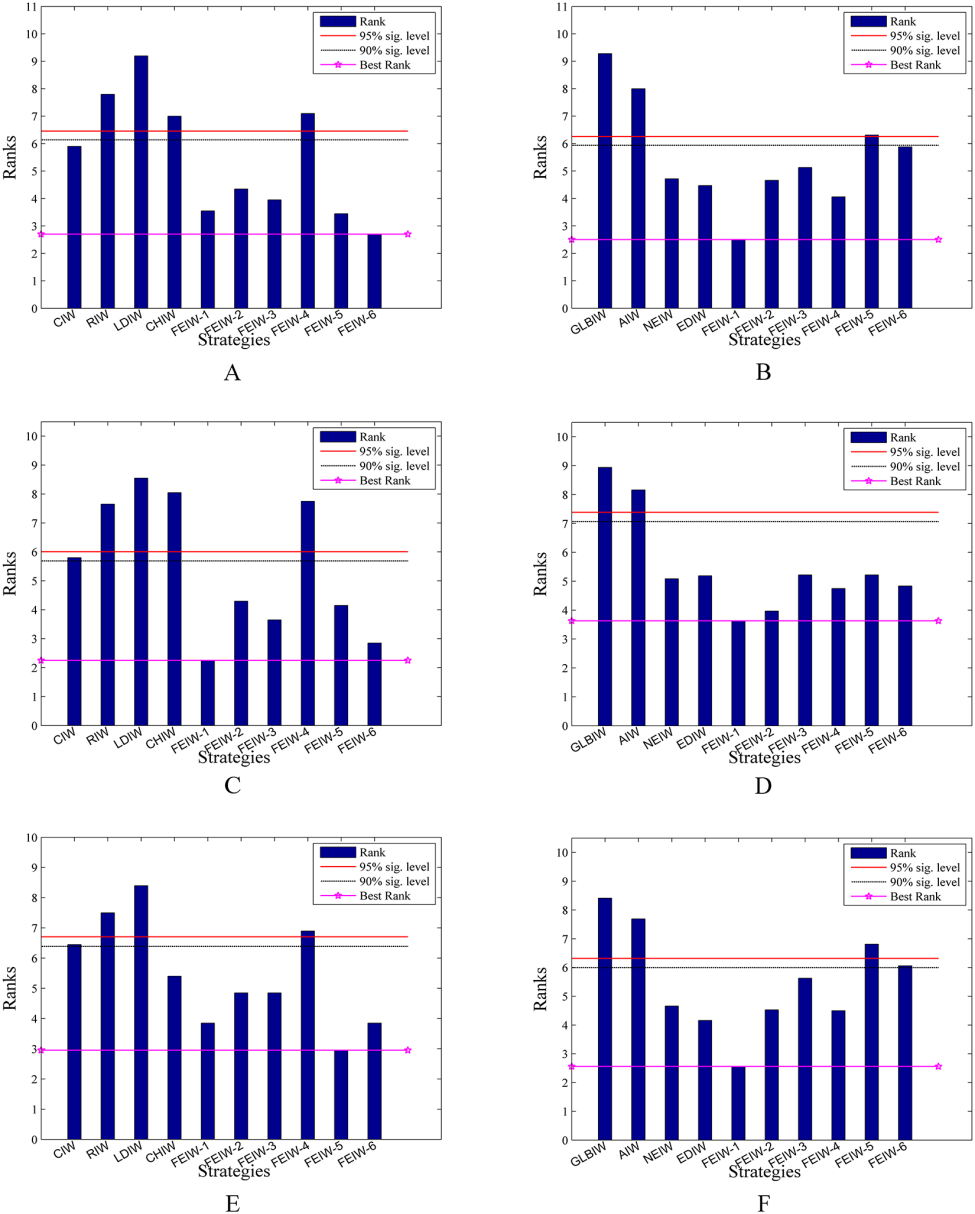
Bonferroni-Dunn bar chart. (A) Average error based on Tables 7 and 8. (B) Average error based on Tables 9 and 10. (C) Minimum error based on Tables 7 and 8. (D) Minimum error based on Tables 9 and 10. (E) Standard deviation of error based on Tables 7 and 8. (F) Standard deviation of error based on Tables 9 and 10.

**Fig 5:**
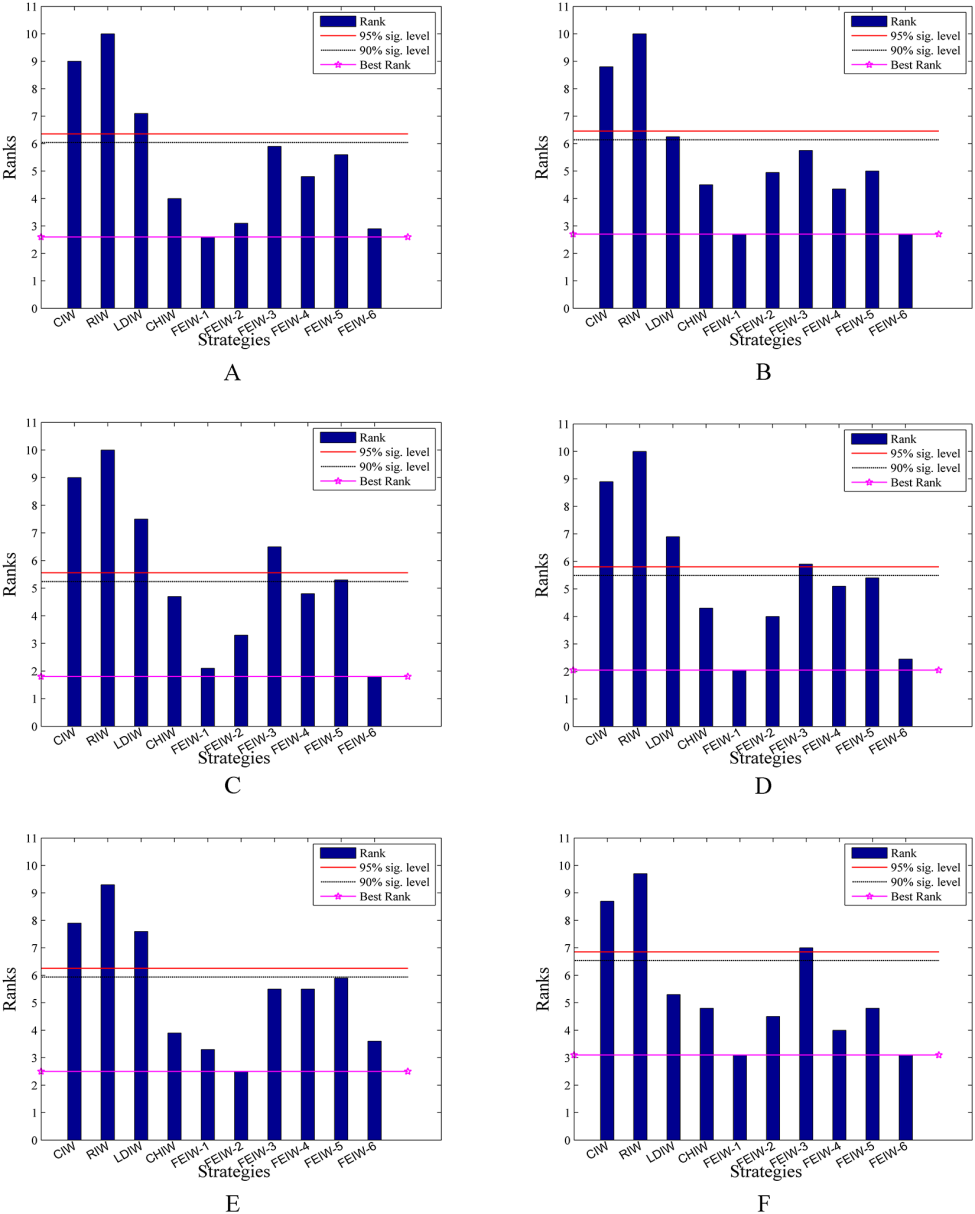
Bonferroni-Dunn bar chart. (A) Average error based on Tables 11 and 12. (B) Average error based on Tables 13 and 14. (C) Minimum error based on Tables 11 and 12. (D) Minimum error based on Tables 13 and 14. (E) Standard deviation of error based on Tables 11 and 12. (F) Standard deviation of error based on Tables 13 and 14.

The difference among mean ranking of PSO variants is illustrated by Bonferroni-Dunn’s graph in Figs 3–5. In Bonferroni-Dunn’s graph, we have drawn a horizontal star-line which represents the threshold for the best performing algorithm (the one with the lowest ranking bar in minimization problems) for a better comparison of variants. A line is drawn for each level of significance considered in this study, at a height equal to the sum of minimum ranking and the corresponding CD computed by the Bonferroni-Dunn method. The bars exceeded these lines are associated to an algorithm having worst performance. In Fig 3, Bonferroni-Dunn bar charts for average and minimum iterations prove that FEIW-1 has the best speed in obtaining the global optimum among all considered IW strategies. Also CIW, RIW, LDIW, CHIW, GLBIW, AIW, NEIW, EDIW, FEIW-2 and FEIW-4 have the worst convergence speed. For success rate criteria, RIW and GLBIW come as worst performers and FEIW-1 and FEIW-5 emerge as best performers. Based on Figs 4 and 5, the other analytical observations are as:

For average error criteria, CIW, RIW, LDIW, CHIW, GLBIW, AIW, FEIW-4 and FEIW-5 emerge as worst performers and FEIW-1 and FEIW-6 as best performers; For minimum error criteria, CIW, RIW, LDIW, CHIW, GLBIW, AIW, FEIW-3 and FEIW-4 come as worst performers and FEIW-1 and FEIW-6 as best performers. For standard deviation criteria, CIW, RIW, LDIW, GLBIW, AIW, FEIW-3, FEIW-4 and FEIW-5 emerge as worst performers and FEIW-1 and FEIW-2 as best performers. Therefore, in general manner, Bonferroni-Dunn bar charts show that FEIW-1 strategy has the best performance among all considered strategies.

#### 6.3.4 Boxplot

**Fig 6:**
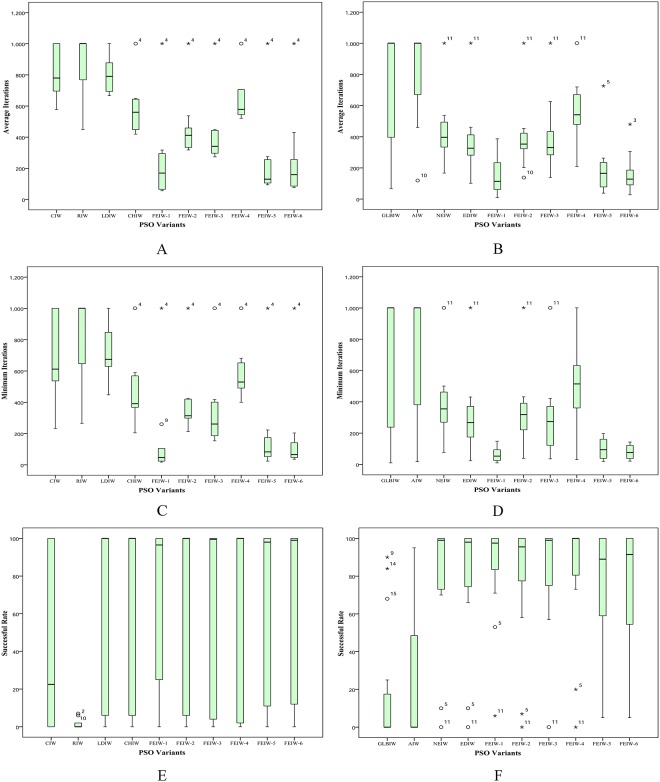
Boxplots of considered PSO variants. (A) Average iterations based on Table 5. (B) Average iterations based on Table 6. (C) Minimum iterations based on Table 5. (D) Minimum iterations based on Table 6. (E) Success rate based on Table 5. (F) Success rate based on Table 6.

**Fig 7:**
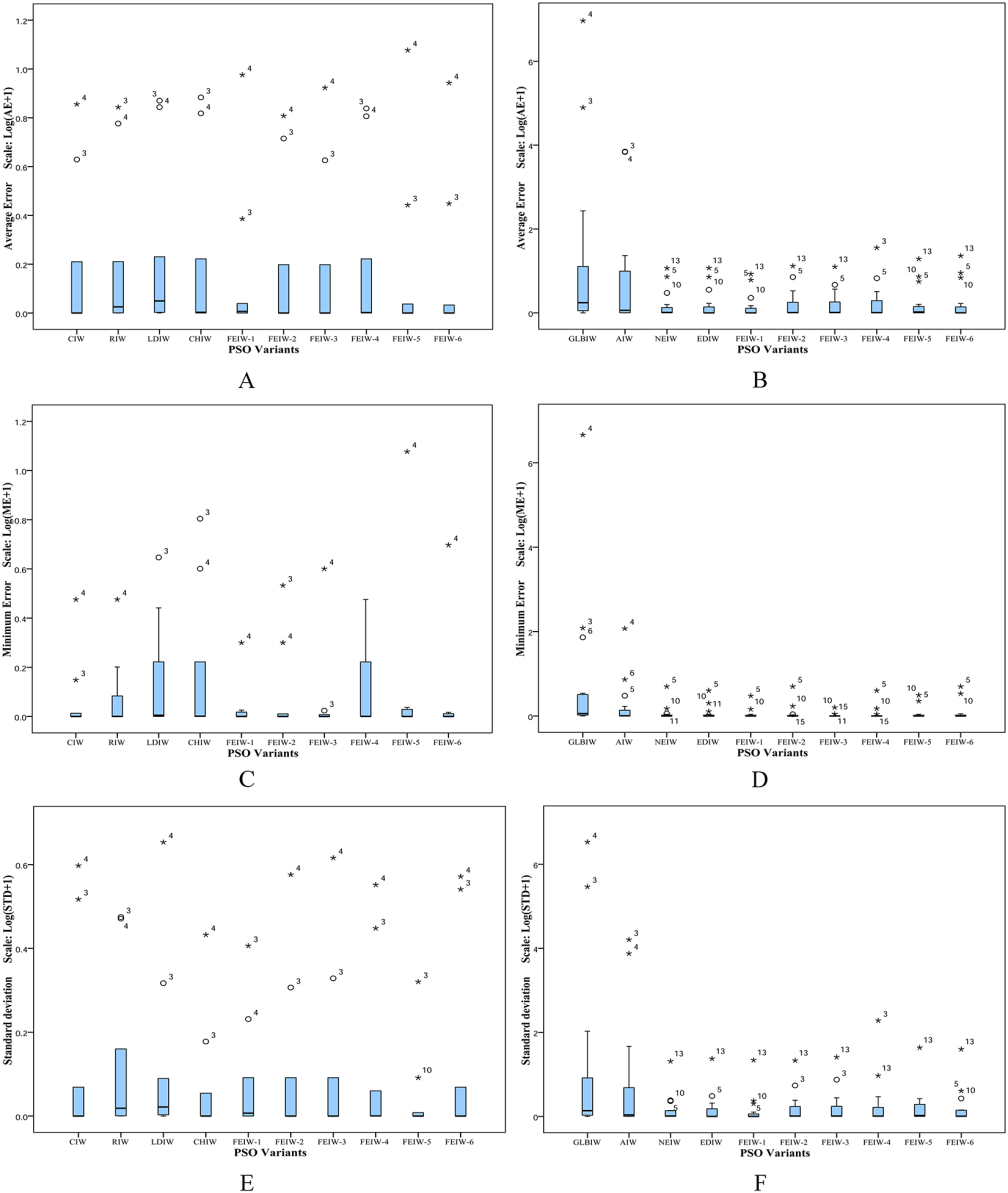
Boxplots of considered PSO variants. (A) Average error based on Tables 7 and 8. (B) Average error based on Tables 9 and 10. (C) Minimum error based on Tables 7 and 8. (D) Minimum error based on Tables 9 and 10. (E) Standard deviation of error based on Tables 7 and 8. (F) Standard deviation of error based on Tables 9 and 10.

**Fig 8:**
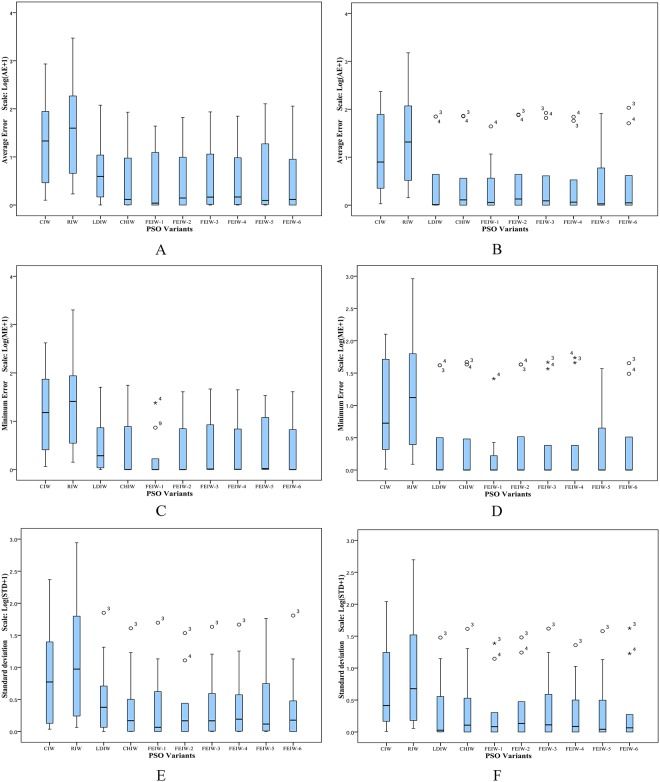
Boxplots of considered PSO variants. (A) Average error based on Tables 11 and 12. (B) Average error based on Tables 13 and 14. (C) Minimum error based on Tables 11 and 12. (D) Minimum error based on Tables 13 and 14. (E) Standard deviation of error based on Tables 11 and 12. (F) Standard deviation of error based on Tables 13 and 14.

In addition to using statistical tests to observe the performance of considered PSO variants, boxplot analysis is also performed for benchmark functions and shown in Figs 6–8. In Fig 6, boxplots of average and minimum iterations show that medians of FEIW-1, FEIW-5 and FEIW-6 are smaller than others. Thus these boxplots show that FEPSO is faster than CIWPSO, RIWPSO, LDIWPSO, CHIWPSO, GLBIWPSO, AIWPSO, NEIWPSO and EDIWPSO. The results of boxplots of average and minimum error in Figs 7 and 8, indicate the superiority of FEIW-1, FEIW-5 and FEIW-6 strategies over other approaches in terms of accuracy. These boxplots prove that FEIW strategy is a reliable IW and has better performance than other considered IW strategies.

### 6.4 Convergence graph

**Fig 9:**
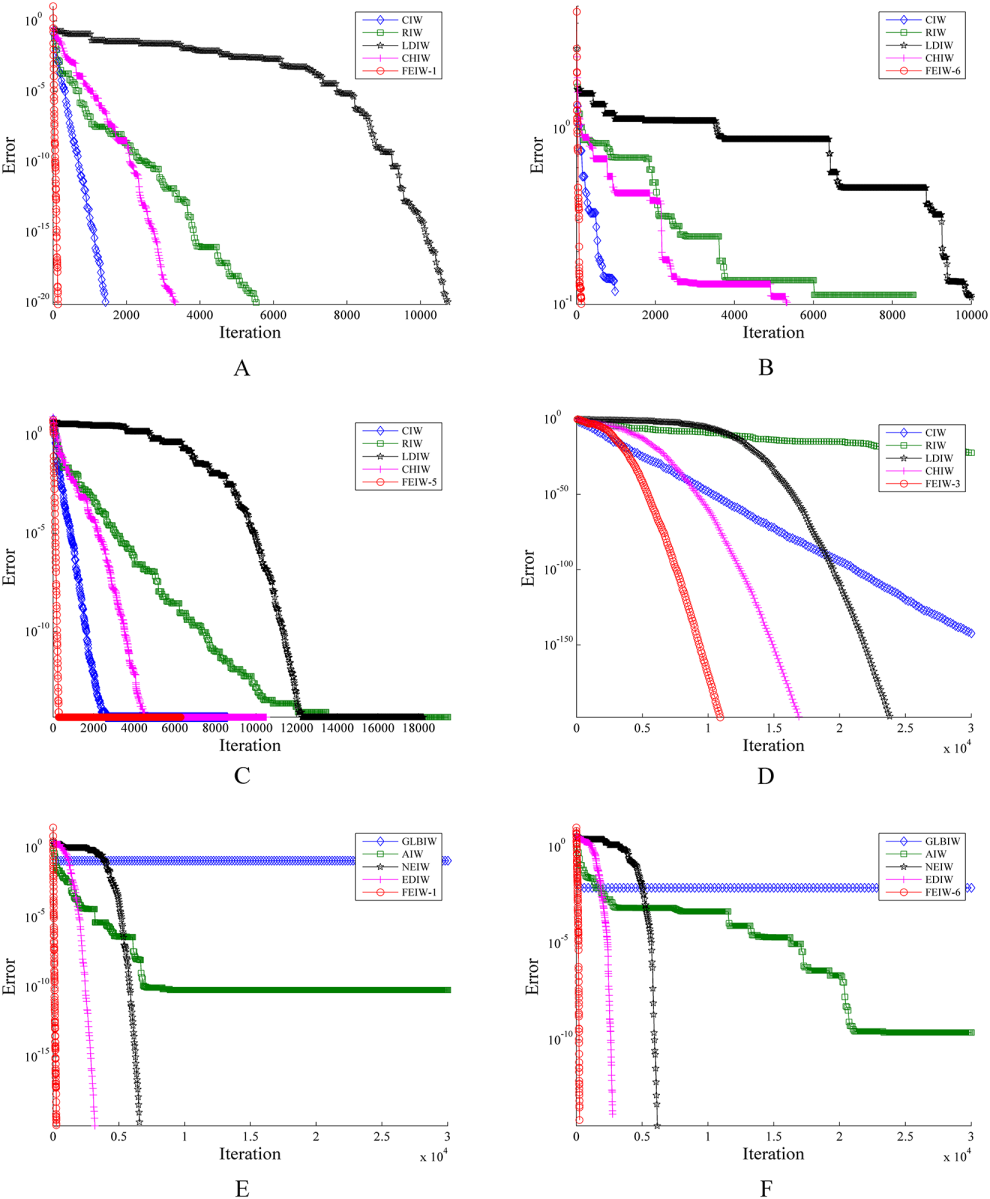
Convergence graph for some PSO variants. (A) Sphere Function with *ε* = 10^−20^. (B) Griewank Function with *ε* = 10^−1^. (C) Ackley Function with *ε* = 10^−15^. (D) Zakharov Function with *ε* = 10^−200^. (E) Schwefel's Problem 2.22 with *ε* = 10^−20^. (F) Weierstrass Function with *ε* = 10^−30^.

The convergence graph for FEIW-1, FEIW-3, FEIW-5 and FEIW-6 is demonstrated in Fig 9. The termination criterion for these graphs is condition 2, where *D* = 10 and *I*_max_ = 30000. From convergence graph, we can discover that the convergence rate of the mentioned IW strategies is clearly faster than the other strategies on the benchmark functions. At the same time, the best solution get by FEPSO is more optimum than by CIWPSO, RIWPSO, LDIWPSO, CHIWPSO, GLBIWPSO, AIWPSO, NEIWPSO and EDIWPSO.

## 7 Conclusion

There are many modifications have been done to the standard PSO algorithm. Some of modifications to the basic PSO are directed towards introducing new strategies of inertia weight which tuned based on trial and error. Suitable selection of the inertia weight provides a balance between global and local searching. This paper proposed a new flexible exponential time-varying inertia weight (FEIW) strategy to improve the performance of PSO. The algorithm named as FEPSO is proposed based on FEIW strategy. We confirmed the FEPSO’s validity in terms of convergence speed and solution precision by testing it with a suit of well-known standard benchmark unimodal and multimodal functions and by comparing obtained results with eight inertia weight strategies of the best time-varying, adaptive and primitive inertia weight strategies. The comparisons are made in terms of convergence speed and solution accuracy and the results are tabulated and graphs are plotted for dimensions 10 and 50 separately. Statistical tests show that this novel strategy converges faster than others during the early stage of the search process and provide better results for problems. Thus experimental results clearly prove the superiority of the proposed model over other inertia weight models. The future work includes the implementation of the FEPSO to solve a real world problem with lots of complexity such as brain MR image segmentation to compare the efficiency of the FEPSO with other recent optimization techniques.
